# Finding the Right Blend: Interplay Between Structure and Sodium Ion Conductivity in the System Na_5_AlS_4_–Na_4_SiS_4_

**DOI:** 10.3389/fchem.2020.00090

**Published:** 2020-02-18

**Authors:** Sascha Harm, Anna-Katharina Hatz, Christian Schneider, Carla Hoefer, Constantin Hoch, Bettina V. Lotsch

**Affiliations:** ^1^Nanochemistry Department, Max Planck Institute for Solid State Research, Stuttgart, Germany; ^2^Department of Chemistry, Ludwig Maximilian University of Munich, Munich, Germany; ^3^Cluster of Excellence E-Conversion, Garching, Germany

**Keywords:** solid-state electrolyte, sodium ion conductor, sulfide, electrochemical impedance spectroscopy, conduction pathway, bond valence energy landscape, Sodium Solid Electrolytes Na_5_AlS_4_-Na_4_SiS_4_

## Abstract

The rational design of high performance sodium solid electrolytes is one of the key challenges in modern battery research. In this work, we identify new sodium ion conductors in the substitution series Na_5-*x*_Al_1-*x*_Si_*x*_S4 (0 ≤ *x* ≤ 1), which are entirely based on earth-abundant elements. These compounds exhibit conductivities ranging from 1.64 · 10^−7^ for Na_4_SiS_4_ to 2.04 · 10^−5^ for Na_8.5_(AlS_4_)_0.5_(SiS_4_)_1.5_ (*x* = 0.75). We determined the crystal structures of the Na^+^-ion conductors Na_4_SiS_4_ as well as hitherto unknown Na_5_AlS_4_ and Na_9_(AlS_4_)(SiS_4_). Na^+^-ion conduction pathways were calculated by bond valence energy landscape (BVEL) calculations for all new structures highlighting the influence of the local coordination symmetry of sodium ions on the energy landscape within this family. Our findings show that the interplay of charge carrier concentration and low site symmetry of sodium ions can enhance the conductivity by several orders of magnitude.

## 1. Introduction

In recent years, all-solid-state batteries (ASSB) have garnered attention as promising candidates for future battery applications in large scale mobility systems, such as electric vehicles (Goodenough, 2012; Janek and Zeier, 2016; Kato et al., 2016). This is due to safety issues arising from liquid electrolytes applied in conventional lithium-ion batteries. Implementing solid electrolytes is thought to provide a more stable battery system, both thermally and mechanically. ASSBs can even pair this advantage with improved energy density through the use of Li or Na metal anodes and bipolar stacking. In prospect, the application of sodium containing materials produced from cheap and abundant sources could effectively cut down costs of ASSBs, thus enabling large-scale energy storage system solutions independent of limited lithium resources (Yabuuchi et al., 2014). One central component of an ASSB is the solid electrolyte. To be applicable for battery systems, the implemented solid electrolytes are required to show high ionic and low electronic conductivity, along with high electrochemical and structural stability, as well as low production costs (Lotsch and Maier, 2017). Regarding conductivity, the group of sulfides includes some of the best solid electrolytes to date. Especially thiophosphates, e.g., Li_10_GeP_2_S_12_ (LGPS), Li_6_PS_5_X (X = Cl, Br, I), and Na_3_PS_4_, are promising materials due to their high ionic conductivities and soft mechanical nature enabling cold pressing of the electrolyte instead of high temperature sintering (Jansen and Henseler, 1992; Kamaya et al., 2011; Rao and Adams, 2011; Hayashi et al., 2012; Kuhn et al., 2013b, 2014; Holzmann et al., 2016). These high ionic conductivities compared to most oxide solid electrolytes are supposed to stem from the high polarizability of the sulfide or thiophosphate anion lattice, facilitating Li or Na ion hopping in the bulk (Wakamura, 1997; Bachman et al., 2016). However, Zeier et al. showed that a softer lattice cannot only lower the migration barrier for charge carriers, but also affects the entropy of migration, which can negatively influence the overall ionic conductivity (Kraft et al., 2017; Krauskopf et al., 2017). This “softness” of the lattice is commonly tuned by isovalent or aliovalent substitution to obtain materials with even higher ionic conductivities. Isovalent substitution is typically employed to introduce softer, more polarizable anions, and to widen diffusion pathways as was studied recently for the solid electrolyte Na_3_PS_4_. In its “cubic” phase, Na_3_PS_4_ exhibits a room temperature ionic conductivity of up to 4.6 · 10^−4^ S cm^−1^ (Hayashi et al., 2014; Krauskopf et al., 2018a). By substitution of S with Se, values up to 1.16 · 10^−3^ S cm^−1^ can be achieved for Na_3_PSe_4_ (Zhang et al., 2015; Krauskopf et al., 2017, 2018b). In addition to isovalent substitution, aliovalent substitution can be used to not only alter the polarizability of the lattice and influence the size of diffusion pathways, but also to tune the charge carrier concentration. Similar to the LGPS system (Kamaya et al., 2011; Bron et al., 2013, 2016; Kuhn et al., 2013a,b, 2014; Kato et al., 2014, 2016; Hori et al., 2015a,b; Harm et al., 2019), tetrel elements were employed in the Na_3+*x*_T_*x*_P_1-*x*_S_4_ (*T* = Si, Sn) system to increase the charge carrier density and increase the overall ionic conductivity. The Sn-containing compounds are structurally very similar to the LGPS-like Li_10_SnP_2_S_12_ and show conductivities of 4 · 10^−5^ S cm^−1^ for Na_10_SnP_2_S_12_ and the highest measured sodium ionic conductivity at room temperature for sulfides of 4 · 10^−3^ S cm^−1^ for Na_11_Sn_2_PS_12_ (Bron et al., 2013; Richards et al., 2016; Duchardt et al., 2018). Aliovalent silicon substitution studies were also conducted for the Na_3_PS_4_ phase achieving a maximum conductivity of 7.4 · 10^−4^ S cm^−1^ for a glass ceramic of composition 94(Na_3_PS_4_).6(Na_4_SiS_4_) (Tanibata et al., 2014). The authors showed the presence of two ion conducting, hitherto unknown crystalline phases in this Na_3+*x*_Si_*x*_P_1-*x*_S_4_ system with formal compositions “Na_11_Si_2_PS_12_” and “Na_4_SiS_4_.” However, no structural information was given nor the reason for the large increase in conductivity of the amorphous ball-milled product (σ = 10^−5^ S cm^−1^) compared to the crystalline products (σ = 10^−7^ S cm^−1^). In this work we expand the materials space of sodium thio-ortho-tetrelates and -trielates by aliovalent substitution of Si in the aforementioned Na_4_SiS_4_ by Al, therefore increasing the number of charge carriers and expanding the lattice by a larger cation with reduced charge ($\text{r}\left(\text{S}{\text{i}}_{\text{Tetr}.}^{4+}\right)=0.26\;Å,\text{r}\left(\text{A}{\text{l}}_{\text{Tetr}.}^{3+}\right)=0.39\;Å$) (Shannon, 1976) to enhance sodium ion conductivity. We present the crystal structures of Na_5_AlS_4_, Na_4_SiS_4_, and Na_9_(AlS_4_)(SiS_4_) and investigate their Na^+^-ion migration pathways by bond valence energy landscape (BVEL) calculations. While Na_5_AlS_4_ was mentioned by Brown et al. and Na_4_SiS_4_ was reported recently by Tanibata et al., no crystallographic data have been reported as yet (Brown and Tani, 1987; Tanibata et al., 2014). In this work we map out the ionic conductivity of the aliovalent substitution series Na_5-*x*_Al_1-*x*_Si_*x*_S_4_ (0 ≤ *x* ≤ 1) and show that the conductivities can be significantly enhanced by tuning the charge carrier or defect concentration. Hereby, the more complex structure of Na_8.5_(AlS_4_)_0.25_(SiS_4_)_0.75_ shows a flatter energy landscape and a jump to a higher conductivity by two orders of magnitude (2.04 · 10^−5^ S cm^−1^) compared to the border phases Na_4_SiS_4_ and Na_5_AlS_4_.

## 2. Experimental Section

### 2.1. Synthesis

Stoichiometric amounts of Na_2_S (Alfa Aesar, 99%), Al_2_S_3_ (Alfa Aesar, 99%), Si (ball milled, Alfa Aesar, 99%), and S (Grüssing, sublimed *in vacuo*) were used as starting materials. An excess of 5 wt% sulfur was added to the mixture to ensure an oxidizing atmosphere during the reaction. Samples were prepared by thoroughly mixing and grinding the starting materials in an agate mortar. The resulting fine powders were transferred into glassy carbon crucibles, compacted and sealed under vacuum into quartz glass ampoules. The ampoules were subsequently transferred into a tube furnace and heated at 50 °C h^−1^ to 600 °C and annealed for 3 d. Subsequently, the furnace was turned off. The ampoules were removed from the furnace when the temperature was below 100 °C and transferred to a glovebox. Na_5_AlS_4_ and Na_4_SiS_4_ samples are off-white to yellow powders, probably from excess sulfur. Na_8.5_(AlS_4_)_0.25_(SiS_4_)_0.75_ crystals were colorless cuboids of about 200 μm diameter embedded in an orange amorphous material, presumably solidified sodium polysulfide melt.

### 2.2. Powder X-Ray Diffraction

From all samples powder X-ray diffractograms (PXRDs) were measured on a Stoe STADI P diffractometer [Ge-(111) monochromator, Dectris Mythen 1 K detector] utilizing Mo-K_α1_ or Cu-K_α1_ radiation in Debye-Scherrer geometry. All samples were sealed in glass capillaries with diameters of 0.3–0.5 mm under argon atmosphere in a glovebox. Indexing of PXRD data, structure solution by charge flipping and subsequent Rietveld refinements were carried out with the program Topas Academic v. 5 (Oszlányi and Sütő, 2008; Coelho, 2018).

### 2.3. Single Crystal X-Ray Diffraction

Single crystals of Na_8.5_(AlS_4_)_0.25_(SiS_4_)_0.75_ were isolated under paraffin oil outside the glovebox and sealed in glass capillaries under oil. Single crystal X-ray diffraction (SCXRD) experiments were carried out with a Bruker D8 Quest diffractometer using Mo-K_α_ radiation. Data handling, including a multi-scan absorption correction with the program SADABS, was done utilizing the Bruker Apex 3 software package (Krause et al., 2015). The structure solution and refinement were performed with the programs SHELXS97 and SHELXL97, respectively (Sheldrick, 2008).

### 2.4. Solid-State Nuclear Magnetic Resonance Spectroscopy

Solid-state NMR spectra were measured on a Bruker Avance III 500 instrument at a magnetic field of B_0_ = 11.74 T. Magic-angle spinning (MAS) experiments were performed in zirconia spinners at a spinning speed of 10 kHz using a Bruker 4 mm triple-channel probe. ^27^Al and ^29^Si spectra were referenced indirectly to ^1^H in 0.1% TMS at 0.00 ppm.

### 2.5. Differential Scanning Calorimetry

For differential scanning calorimetry (DSC) measurement samples were sealed in small quartz ampoules (5 mm outer diameter, 10–15 mm length) under argon. To improve heat-flow the quartz ampoules were put in Pt-crucibles (6 mm diameter, 10 mm height). Measurements were then carried out using a Netzsch STA 449 F5 Jupiter with an Argon flow of 40 mL min^−1^ in a temperature range between room temperature and 900 °C and heating/cooling rates between 1 and 10 K min^−1^. Data handling was performed with the Netzsch Proteus software package.

### 2.6. Energy Dispersive X-Ray Analysis

Elemental composition was determined by energy-dispersive X-ray spectroscopy (EDX; detector: Oxford Instruments Inca Energy) and an image of the morphology was obtained using a Jeol JSM 6500 F scanning electron microscope (SEM; field emission gun, acceleration voltage 20 kV).

### 2.7. Bond Valence Energy Landscape Calculations

Bond valence energy landscape (BVEL) calculations were performed with the program 3Dbvsmapper (Sale and Avdeev, 2012). The BV method calculates the bond valence sum (BVS) for a tested ion at each voxel grid point of a three-dimensional mesh in a unit cell. For a sodium ion at its equilibrium site relative to the other ions in the structure (often equal to the crystallographic site of the sodium ion) the bond valence sum should be equal to its oxidation state (+1). Deviations of the BVS display possible migration pathways for the tested ion (Nishitani et al., 2018). For a detailed description of the method see the Supplementary Information. Here, the BVEL method uses soft-bond-valence parameters and additional (penalty) terms to account for Coulombic attraction/repulsion terms (Adams and Rao, 2011) The cutoff distance was fixed to a maximum value of 8 Å. The images were created with VESTA (Momma and Izumi, 2011).

### 2.8. Electrochemical Impedance Spectroscopy

Electrochemical impedance spectroscopy and galvanostatic polarization measurements were performed with an Ivium compactstat.h (24 bit instrument) in a two-electrode setup using a rhd Instruments Microcell HC cell stand loaded with rhd Instruments TSC Battery cells performing measurements between 25 and 75°C inside the glovebox under argon atmosphere. The spectra were recorded in a frequency range of 1 MHz–0.1 Hz and an applied rms AC voltage between 30 and 100 mV. The analysis of the impedance spectra was carried out with the RelaxIS3 software from rhd Instruments. The linearity, stability and causality was checked by applying the Kramers-Kronig-relation before fitting the data. Before measuring, the samples were ground thoroughly and compacted to a pellet of about 0.5 mm thickness and 5 mm diameter by uniaxial cold pressing (500 MPa). The obtained densities of the pellets were between 76 and 91% with an error of 6% (cf. Table S13). For impedance spectroscopy, the pellets were sandwiched between indium foil (Alfa Aesar, 0.127 mm thick, 99.99%) to enhance the contact with the stainless steel electrodes of the cells. No reaction between In and the samples was observed. Every sample was measured twice, and for each sample several temperature cycles were conducted. The measurement uncertainties arise from the error propagation of the uncertainties in pellet thickness, area and in obtained resistance. For the galvanostatic polarization measurements stainless steel electrodes were used.

## 3. Results and Discussion

### 3.1. X-Ray Diffraction

**Figure 1:**
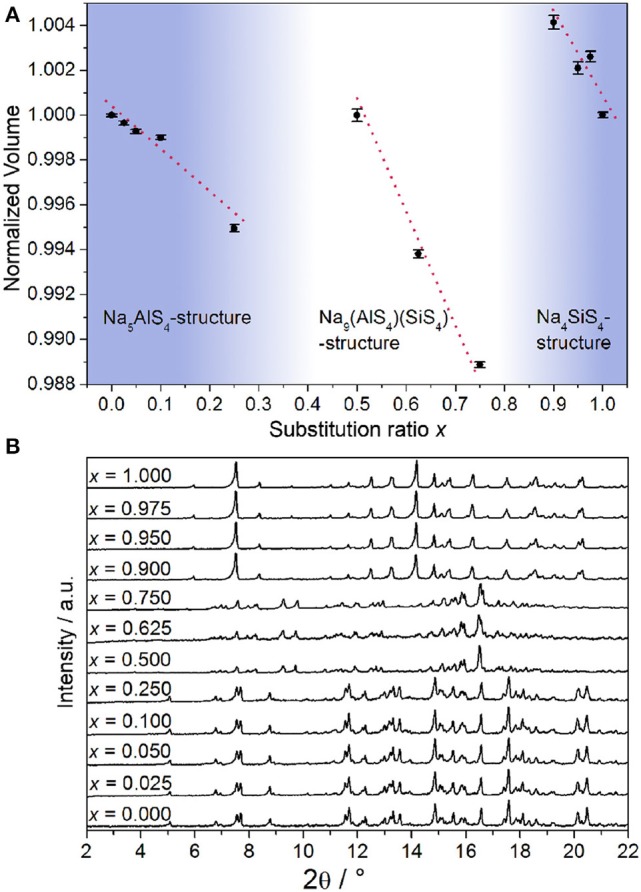
**(A)** Normalized volume of the observed crystalline phases obtained by Rietveld refinement displayed against substitution value *x*; error bars represent 3σ and the red lines are a guide to the eye to illustrate the Vegard-like dependence on *x*. **(B)** PXRDs (Mo-K_α1_ radiation) of all samples in the Na_5-*x*_Al_1-*x*_Si_*x*_S_4_ (0 ≤ *x* ≤ 1) substitution series.

From all samples in the Na_5-*x*_Al_1-*x*_Si_*x*_S_4_ (0 ≤ *x* ≤ 1) aliovalent substitution series PXRDs were measured to study the crystallinity and phase composition (additional crystallographic data for all structures are given in the Supplementary Information). Figure 1B shows that no complete solid solution is formed. Instead, three separate phases crystallize as a function of the degree of substitution *x*. This is consistent with the fact that the pseudo-binary border phases Na_5_AlS_4_ and Na_4_SiS_4_ do not crystallize isotypically as shown below. Regarding the volume of the respective crystalline phases depicted in Figure 1A, a Vegard-like dependence on the substitution value *x* for all phases can be observed and therefore partial miscibility within the respective phases can be assumed (Vegard, 1921).

#### 3.1.1. Crystal Structure of Na_5_AlS_4_

**Figure 2:**
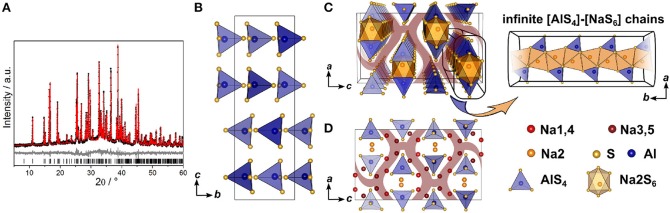
**(A)** Rietveld refinement of Na_5_AlS_4_ (*x* = 0) measured using Cu-*K*_α1_ radiation; black dots depict the measured data, red lines the Rietveld fit, gray lines the difference plot and black lines the respective reflection positions. **(B)** [AlS_4_]-tetrahedral sublattice in Na_5_AlS_4_ viewed along *a*. **(C)** Perspective view of the [AlS_4_]-tetrahedral and [Na2S_6_] octahedral arrangement parallel to *b*. **(D)** Complete Na_5_AlS_4_ structure with [AlS_4_]-tetrahedral arrangement viewed along *b*; maroon curved lines represent sodium ion diffusion pathways determined by BVEL calculations.

Since no suitable single crystals were obtained, the crystal structure of Na_5_AlS_4_ was determined using powder X-ray data. The PXRD of Na_5_AlS_4_ was indexed in the orthorhombic space group *Pbca* (No. 61) with *a* = 12.0130(12) Å, *b* = 7.052 63(7) Å, and *c* = 21.5605(2) Å. The structure was solved by charge-flipping implemented in Topas Academic v.5 and refined with the Rietveld algorithm (Figure 2A). The structure is depicted in Figure 2. The compound crystallizes in the Na_5_FeO_4_ structure type and is composed of isolated [AlS_4_]-tetrahedra and distorted [NaS_4_]-tetrahedra and [NaS_6_]-octahedra (Brachtel and Hoppe, 1978). The packing of the Al^3+^ atoms and therefore the packing of the (AlS_4_)^5−^-anions can be regarded as a slightly distorted α-uranium packing as was stated for isotypic Rb_5_GaO_4_ (Bender et al., 2010). The BVEL calculations (cf. below) show that most likely the Na2 atom does not take part in the sodium ion conduction and can therefore be considered as being part of the lattice. Hence, the lattice can be regarded as hexagonally packed infinite chains of face-sharing [Na2S_6_]-octahedra connected via a common face to [AlS_4_]-tetrahedra which alternate back and forth along *a* (Figure 2C).

#### 3.1.2. Crystal Structure of Na_4_SiS_4_

**Figure 3:**
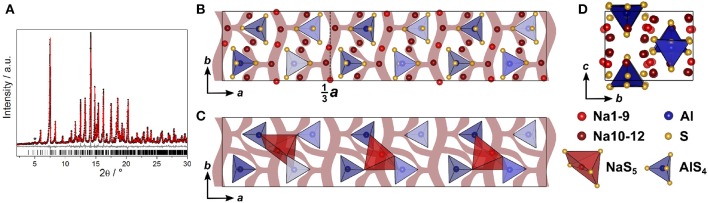
**(A)** Rietveld refinement of Na_4_SiS_4_ (*x* = 1) measured using Mo-*K*_α1_ radiation, the super-structure reflection is marked by an asterisk; black dots depict the measured data, red lines the Rietveld fit, gray lines the difference plot and black lines the respective reflection positions. **(B)** Na_4_SiS_4_ crystal structure parallel to *c*; **(C)** Na_4_SiS_4_ structure viewed along *a*; **(D)** [SiS_4_]-tetrahedra (blue) and [NaS_5_]-pyramidal (red) arrangement viewed parallel to *c*, showing the [NaS_5_]-pyramids in a one-up-two-down-pattern; maroon curved lines represent sodium ion diffusion pathways determined by BVEL calculations.

The structure of Na_4_SiS_4_ was also determined from PXRD data. The diffractogram was indexed in the orthorhombic space group *P*2_1_2_1_2_1_ (No. 19) with *a* = 13.6765(3) Å, *b* = 8.7839(2) Å, and *c* = 6.889 40(15) Å, solved using charge-flipping and refined by Rietveld refinement (Figure 3A). The structure is comprised of isolated [SiS_4_]-tetrahedra which are edge- and corner-sharing to distorted [NaS_6_]-octahedra (5+1 coordination, cf. below). The sulfur atom arrangement constitutes a distorted hexagonal close packing (hcp). Therefore, the structure can be regarded as a hcp of S^2−^-anions with Si^4+^ and Na^+^ filling $\frac{1}{8}$ tetrahedral and all octahedral voids, respectively. This highlights the similarity of this compound's structure with the thio-LiSICON family (Lotsch and Maier, 2017). However, this structure model does not account for the weak reflection at 2Θ ≈ 5°, marked in Figure 3A. It stems from an elongation of the *a*-axis by a factor of three (*i*3 transition) to *a* = 41.0301(7) Å and an ordering of sodium atoms Na10, Na11, and Na12 to form [NaS_5_]-pyramids in a one-up-two-down-pattern, leading to the superstructure shown in Figure 3.

#### 3.1.3. Crystal Structure of Na_9_(AlS_4_)(SiS_4_)

**Figure 4:**
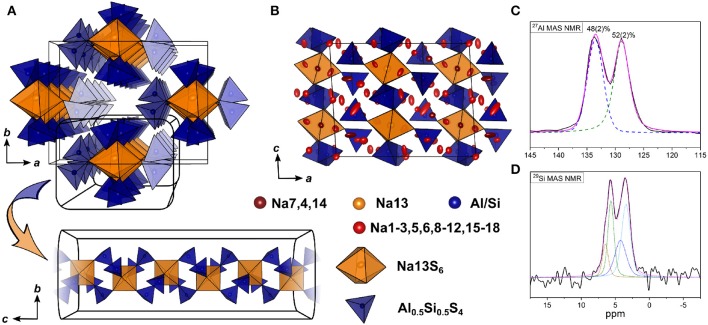
**(A)** [Si/AlS_4_]-tetrahedral and [Na13S_6_]-octahedral arrangement viewed along *c* and infinite [Si/AlS_4_]-[Na13S_6_]-rods viewed parallel to *a*. **(B)** Na_9_(AlS_4_)(SiS_4_) crystal structure viewed along *b*; blue polyhedra depict [Si/AlS_4_]-tetrahedra, orange polyhedra depict [Na13S_6_]-octahedra, red, dark red, and yellow atoms depict Na, and blue atoms depict Si/Al; ellipsoids were drawn at 80% probability. **(C)** Deconvoluted ^27^Al MAS NMR spectrum of Na_9_(AlS_4_)(SiS_4_) (*x* = 0.5); purple line shows the overall fit, colored dashed lines represent the contributing pseudo-Voigt peaks, relative intensities are given with their respective standard deviation in parentheses. **(D)** ^29^Si MAS NMR spectrum of Na_9_(AlS_4_)(SiS_4_); colored dotted lines show a tentative signal distribution.

The double salt Na_10-2*x*_(AlS_4_)_2-2*x*_(SiS_4_)_2*x*_ could be obtained in a compositional range of 0.5 ≤ *x* ≤ 0.75. Samples with *x* = 0.75 yielded suitable crystals for SCXRD measurements, presumably because a poly-sulfide melt serves as a solvent for the compound at temperatures exceeding 300 °C as shown by thermal analysis (cf. Figure S3). Na_8.5_(AlS_4_)_0.5_(SiS_4_)_1.5_ (*x* = 0.75) crystallizes in the monoclinic space group *Cc* (No. 9), with *a* = 17.5673(6) Å, *b* = 13.5408(5) Å, *c* = 14.2543(5) Å, and β = 93.3683(13) °. Its crystal structure is comprised of isolated [Al/SiS_4_]-tetrahedra, and distorted tetrahedrally, trigonal-bipyramidally, square-pyramidally or octahedrally coordinated [NaS_4_]-, [NaS_5_]-, or [NaS_6_]-units (Figure 4, Figure S1). Additionally, the anion sublattice shows pseudo-inversion symmetry, which is broken by the sodium cations. Since BVEL calculations show that Na13 requires the highest energy to take part in ion migration (cf. below), it can be considered as part of the lattice. Therefore, the topology of the structure can be described as a distorted hexagonal packing of rods comprised of [Na13S_6_] corner-sharing to four [Al/SiS_4_]-tetrahedra and interconnected by two corner-sharing [Al/SiS_4_]-tetrahedra parallel to *c* (Figure 4A). In contrast, for Na cations Na4, Na12, Na15, and Na18 (cf. Table S8) not taking part in the lattice, large anisotropic displacement parameters are found (see Table S9 and Figure S1). They occupy positions best described as two half-filled face-sharing [NaS_4_]-tetrahedra constituting an unresolved split position, which therefore explains the elongated shapes. The occupancy of Si vs. Al was not refined because of the similar atomic form factors of both elements, yet the Si/Al ratio was confirmed to be 3/1 by EDX measurements (cf. Table S11). The mean Al/Si–S-distances (cf. Table S10) of all four atomic sites of 2.147(3) Å (Si1S_4_), 2.165(3) Å (Si2S_4_), 2.166(3) Å (Si3S_4_), and 2.160(3) Å (Si4S_4_) are in between the distance expected for tetrahedrally coordinated Si^4+^–S of 2.10 Å and Al^3+^–S of 2.23 Å (Shannon, 1976). This suggests that all Al/Si sites are occupied by silicon and aluminum with Si1 having a slightly higher Si/Al ratio than the other three sites. Since the single crystal was obtained from a sample with *x* = 0.75, the occupancy of sodium atoms was expected to be <1 to maintain charge neutrality. Therefore, the occupancy of all sodium atoms was freely refined (cf. Table S8) insofar as their occupancy factor was significantly (≥ 3σ) lower than one, yielding a total number of sodium atoms per unit cell of 66.9(2), which is in good agreement with the nominal value of 68.

### 3.2. NMR Spectroscopy

To verify the assumption of a mixed occupancy of all four atomic Al/Si sites in the compound Na_9_(AlS_4_)(SiS_4_), ^27^Al, and ^29^Si magic-angle spinning (MAS) NMR spectra were collected (Figures 4C, D). Both spectra show two clearly separated peaks with noticeable shoulders, especially in the ^29^Si spectrum. Although four signals in each spectrum are expected due to the four crystallographically independent Al/Si sites, the occurrence of only two peaks in each spectrum is in good agreement with the crystal structure by taking into account that the [Al/SiS_4_] sub-lattice shows pseudo-inversion symmetry and therefore the chemical shifts of the respective nuclei should be very similar (or accidentally equal), resulting in two sets of two overlapping signals, which is apparent in the ^29^Si spectrum and, to a lesser extent, also in the ^27^Al spectrum. Additionally, the appearance of shoulders in the spectra suggests slightly different Si/Al occupancies for the atomic sites with pseudo-inversion symmetry, which is also corroborated by the mean Al/Si–S distances from SCXRD data.

### 3.3. Bond Valence Energy Landscape Calculations

BVEL calculations were performed in order to elucidate the minimum energy trajectories of the sodium ions and their dimensionalities in the three structures Na_5_AlS_4_, Na_9_(AlS_4_)(SiS_4_), and Na_4_SiS_4_. The bond valence approach was proven to be a valid starting point for discussing ion migration pathways in crystalline (ionic) solid electrolytes and electrode materials. The method provides reasonable pathways, comparable to those obtained by density functional theory (DFT) or molecular dynamics (MD) simulations (Avdeev et al., 2012; Xiao et al., 2015). During ion migration (here Na^+^) from one equilibrium site Na_i_ (often a crystallographic site) to an adjacent site Na_j_, sodium surpasses one (or multiple) transition state(s). Meta-stable sites along the path are considered to be interstitial sites for sodium ions. In this work, we denote the calculated global minimum energy ${\text{E}}_{\text{min}}^{\text{global}}$, the minimum energy within the infinitely connected pathway ${\text{E}}_{\text{min}}^{\text{path}}$ and the energy at which a infinitely connected pathway is formed ${\text{E}}_{\text{mig}}^{\text{path}}$. The energy required for overcoming the ion migration barrier height Δ is calculated by $\Delta \text{E}=|{\text{E}}_{\text{min}}^{\text{path}}-{\text{E}}_{\text{mig}}^{\text{path}}|$. Subscript abbreviations denote the dimensionality of the pathway. Keeping in mind, that these calculated barrier heights for ion migration are overestimated, due to not taking lattice relaxations and Coulombic repulsion of Na^+^–Na^+^ into account, the BV method provides elucidating insights into probable ion migration pathways in a new structure.

#### 3.3.1. BVEL Calculations for Na_5_AlS_4_

**Figure 5:**
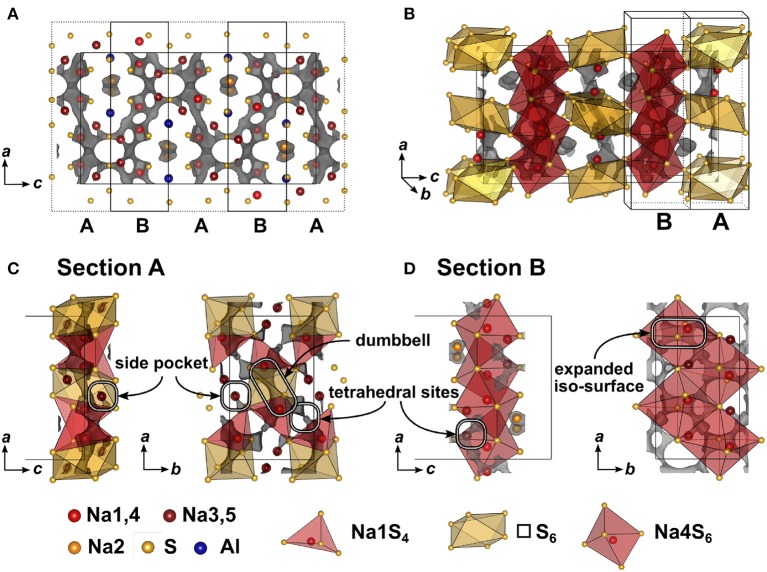
**(A)** Na_5_AlS_4_ crystal structure viewed along *b* direction. Bond valence energy landscape at isoenergy value of −2.40 eV ($E_{min}^{global}=-3.71\;eV$, $E_{min}^{path}=-3.43\;eV$, $E_{mig}^{path}=-2.58\;eV$,Δ*E*_3*D*_ = 0.83 eV). The unit cell is divided along the crystallographic *c* direction into alternating sections **A** and **B**. **(B)** Na_5_AlS_4_ unit cell with sections **A** and **B**. **(C)** Section **A** (*c* = 0.9 − 1.1) viewed along *b* and *c*. **(D)** Section **B** (*c* = 0.6 − 0.9) viewed along *b* and *c*.

In Figure 5 the result of the BVEL calculation of Na_5_AlS_4_ is depicted. To better decipher the individual, spatially distinct components of the overall Na ion trajectory, we introduce sections **A** and **B** in Figure 5A, and separately discuss each section. In section **A** tetrahedrally coordinated Na1 form a 2D-like conduction pathway in the *ac* plane. Two adjacent [Na1S_4_] tetrahedra are connected via shared faces of an [□ S_6_] octahedron, creating a dumbbell-like conduction network between two Na1 sites as shown in Figure 5C. Unoccupied tetrahedral sites (see Figure 5C) loosely connect the Na1–Na1 dumbbells. Each of these dumbbells is connected to two Na3, which are residing in peninsular-like side pockets. Section **B** comprises a two-dimensional network composed of larger areas of low sodium ion bond valence energy (expanded isosurface) connected via unoccupied tetrahedral sites. These rectangular shaped areas, residing inside an octahedral cavity created by six sulfide ions (red octahedron, Figure 5D), display regions in which sodium can migrate freely without passing through high energy bottlenecks. This region is visible at low bond valence isoenergy of −3.0 eV in Figure S4. Na4 resides in one of the corners of the expanded isosurface, thus creating a [Na4S_6_] coordination polyhedron. The [Na4S_6_] octahedra are connected via unoccupied tetrahedral sites forming a percolating network. The infinite [AlS_4_]-[NaS_6_] chains obstruct ion conduction along the crystallographic *c* direction, but allow connection of both sections **A** and **B** at the gap between two chains as depicted with the maroon colored curved lines in Figures 2C,D forming a zig-zag pattern along *c* and a two-dimensional pattern in the *ab* plane. Consequently, despite its more dominant 2D conduction pathways in *ab* plane, Na_5_AlS_4_ is expected to be a three-dimensional ion conductor.

#### 3.3.2. BVEL Calculations for Na_4_SiS_4_

**Figure 6:**
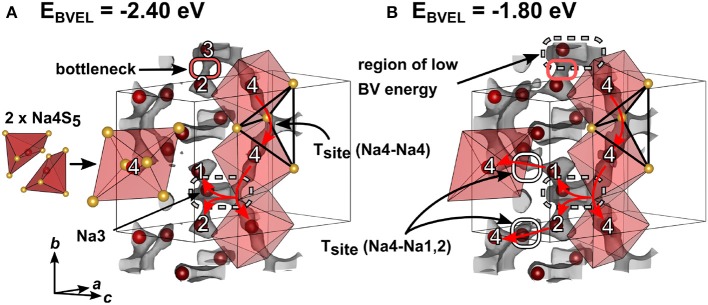
Crystal structure of Na_4_SiS_4_ (simplified model without superstructure) with sodium atoms drawn in red and sulfur atoms drawn in yellow. Bond valence energy landscape at isoenergy values of **(A)** −2.40 eV and **(B)** −1.80 eV are drawn in gray ($E_{min}^{global}=-3.73\;eV$, $E_{min}^{path}=-3.73\;eV$, $E_{mig}^{path}=-2.68\;eV,\Delta E_{2D}=1.05\;eV$). Red arrows depict sodium ion diffusion pathways. Numbers denote crystallographic sodium sites.

For simplification, the orthorhombic structure with a shorter *a* axis in Figure S5 instead of the superstructure of Na_4_SiS_4_ in Figure 3 was used to calculate the bond valence energy landscape for Na_4_SiS_4_. This does not lead to an appreciably different BVEL outcome, since the superstructure is a result of sodium atom ordering. The anionic lattice remains the same in both structure models. Figure 6 depicts the structure of Na_4_SiS_4_ together with bond valence energy surfaces of different isoenergy values. A more detailed illustration of the evolution of BVE isosurfaces can be found in Figure S5. As depicted in Figure 6, Na4 occupies a distorted square pyramid. Two base-sharing pyramids form a larger octahedron with Na4 preferentially occupying one half of the octahedron. In a short range, hopping through the common base of two adjacent square pyramids is energetically facile for Na4. For long-range diffusion, Na4 can hop via a tetrahedral site spanned by two [Na4S_6_] units into an expanded region of low bond valence energy (up or down along *b*). Na3 resides close to one of the [Na4S_6_] unit's corners. Despite being connected to this low energy site as well, Na1 and Na2 have to pass a bottleneck when diffusing to this site. Therefore, mainly Na4 and Na3 form a quasi-one-dimensional, channel-like structure in *b* direction. At slightly higher bond valence energies tetrahedral sites between Na4-Na1 and Na4-Na2 are accessible through small bottlenecks (see Figure 6B, gray isosurface marked with red ellipses). The resulting network percolates the unit cell in all crystallographic directions, resulting in 3D ion migration at higher energies (Δ_3D_ ≈ 1.6 eV).

#### 3.3.3. BVEL Calculations for Na_9_(AlS_4_)(SiS_4_)

**Figure 7:**
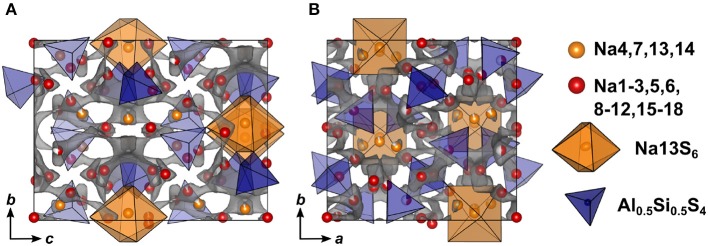
Crystal structure of Na_9_(AlS_4_)(SiS_4_) along *a* **(A)** and *c* **(B)** direction. The bond valence energy landscape at isoenergy value of −2.60 eV is drawn in gray ($E_{min}^{global}=-4.02\;eV$, $E_{min}^{path}=-3.86\;eV$, $E_{mig}^{path}=-2.64\;eV$, Δ*E*_3*D*_ = 1.22 eV).

Compared to Na_5_AlS_5_ and Na_4_SiS_4_, the double salt Na_9_(AlS_4_)(SiS_4_) is structurally more complex, since it features 18 sodium sites hosted in mostly distorted coordination polyhedra. Only Na13 resides in a rather ordered [Na13S_6_]-octahedron, which is bridged in *c* direction by corner-sharing [AlS_4_]^5−^/[SiS_4_]^4−^-tetrahedra (cf. Figures 4, 7).

In terms of conduction pathways, the sodium ions in Na_9_(AlS_4_)(SiS_4_) can be divided into isolated and migrating sodium ions. Migrating sodium ions, depicted as red spheres in Figure 7, reside inside the calculated BVEL network at isoenergy ${\text{E}}_{\text{mig}}^{\text{path}}$. Most of the sodium ions contributing to the three-dimensional conduction are mainly square-pyramidally coordinated, but also trigonal bipyramidal, tetrahedral or octahedral coordinated. Presumably, the low local coordination symmetry of those sodium ions and the therefore asymmetric charge distribution leads to less Coulombic attraction facilitating ion hopping. However, the either trigonal bipyramidally (Na4,7), tetrahedrally (Na14), or octahedrally (Na13) coordinated sodium ions are not connected to the conduction network at ${\text{E}}_{\text{mig}}^{\text{path}}$ and thus are considered to be members of the rigid framework (cf. Figure S6). At noticeably higher energy all sodium ions but Na13 connect to a large, expanded network. Summarizing, at ${\text{E}}_{\text{mig}}^{\text{path}}$ a very flat three-dimensional isoenergy surface with only a few bottle necks occupies a large volume fraction of the unit cell. Compared to Na_5_AlS_4_ and Na_4_SiS_4_, in terms of conduction pathways, the sodium ions in Na_9_(AlS_4_)(SiS_4_) are expected to show higher mobility due to higher versatility in the sodium ion coordination and the resulting flatter potential energy surface for sodium ion migration.

### 3.4. Electrochemical Impedance Spectroscopy

**Figure 8:**
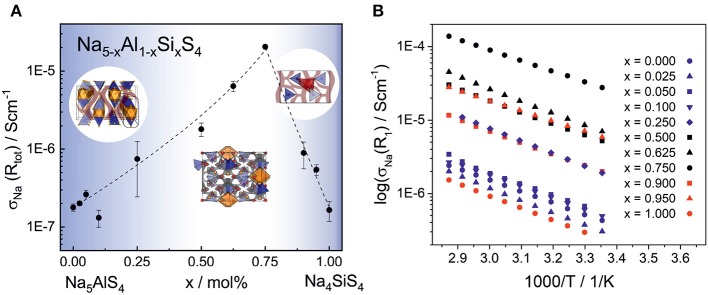
**(A)** Sodium ion conductivity in the phase system Na_5-*x*_Al_1-*x*_Si_*x*_S_4_ with (0 ≤ *x* ≤ 1) as a function of the substitution factor *x* (visualized by dashed line). In the range of *x* = 0.50–0.75, where the material crystallizes in the Na_9_(AlS_4_)(SiS_4_)-structure, the highest average conductivity of 2.04 · 10^−5^ S cm^−1^ was observed. The unsubstituted phases Na_5_AlS_4_ and Na_4_SiS_4_ show a significantly lower ionic conductivity of 1.64 · 10^−7^ -1.77 · 10^−7^ S cm^−1^, respectively. The error bars include the standard deviation of the sample and the error of the measurement of about 8%. **(B)** Temperature dependent sodium ion conductivities calculated from R1 of all phases for a selection of measurements (all parameters for these particular measurements are given in Tables S12, S13). The different colors indicate the different crystal structures: Na_5_AlS_4_-structure (blue), Na_9_(AlS_4_)(SiS_4_)-structure (black), and Na_4_SiS_4_-structure (red).

Electrochemical impedance spectroscopy in the temperature range 25–75°C was conducted on cold pressed samples of all members in the series Na_5-*x*_Al_1-*x*_S*i*_*x*_S_4_ with (0 ≤ *x* ≤ 1). As depicted in Figure 8, they show averaged sodium ion conductivities ranging from 1.64 · 10^−7^ S cm^−1^ for Na_4_SiS_4_ up to 2.04 · 10^−5^ S cm^−1^ for Na_8.5_(AlS_4_)_0.5_(SiS_4_)_1.5_. Galvanostatic polarization measurements (cf. Figure S7) confirm the mainly ion conducting nature of the materials with transference numbers of about 0.9998, thus being suitable as solid electrolyte in a battery. The conductivities in Figure 8 represent the total conductivities of the samples, modeled by a capacitor or constant phase element (CPE) and a resistance (R) in parallel. Where necessary, a second R-CPE-element was added, and in each spectrum the polarization of ions at the interface of the blocking electrode was modeled by a CPE. Exemplary impedance spectra and equivalent circuits of each sample as well as the respective capacitances and ideality factors (α) are given in Figure S8 and Table S12. The effective capacitances (C_eff_) were calculated by the Brug formula (Brug et al., 1984) C_eff_=(R(CPE))^1/α^/R. The best conducting sample Na_8.5_(AlS_4_)_0.5_(SiS_4_)_1.5_ shows only one semicircle with a capacitance of about 2 · 10^−10^ F. The capacitances of the high frequency semicircle of all other samples are of the same order of magnitude, suggesting the same underlying processes. According to literature, the capacitance of 1 · 10^−10^ F corresponds to grain boundary contributions (Irvine et al., 1990). Thus, the high frequency arc contains the information about the bulk and grain boundaries, but the exact bulk contributions can not be deconvoluted. In some spectra a second semicircle at lower frequencies with capacitances of about 8 · 10^−8^ F-6 · 10^−7^ F is present. The activation energies of this process, calculated according to $\sigma =\sigma_{0}/\text{T}\cdot {\text{e}}^{-{\text{E}}_{\text{a}}/{\text{k}}_{\text{B}}\text{T}}$ (with σ_0_ being the pre-factor, E_a_ the activation energy, k_B_ the Boltzmann constant and T the temperature), are higher than the activation energies obtained from the high frequency semicircles (cf. Tables S14, S15). Consequently, this semicircle may stem from an inhomogeneity in composition or an additional resistive layer on the surface (Bruce and West, 1983; Irvine et al., 1990). Interestingly, the low frequency semicircle is absent for the best conducting sample Na_8.5_(AlS_4_)_0.5_(SiS_4_)_1.5_ pointing to an easier handling of this material. To avoid a mingling of processes, only the data from the high frequency semicircle is applied for discussing the trends in activation energy and pre-factor in the following. A plot only including conductivities calculated from the high frequency semicircles is given in Figure S9. It exhibits the same trend as in Figure 8 with Na_8.5_(AlS_4_)_0.5_(SiS_4_)_1.5_ being the best conducting member of the series.

### 3.5. Discussion

Taking a closer look at Figure 8 and Figure S9 reveals the strong influence of the number of charge carriers on the conductivity of each material. Going from the poor ionic conductor Na_5_AlS_4_ along the isotypic phases up to *x* = 0.25, the substitution of Al^3+^ with Si^4+^ introduces sodium vacancies, which increases the conductivity. On the other end of the series, Na_4_SiS_4_ shows a σ_Na_ in line with findings from Tanibata et al. (2014). Here, the amount of sodium ions is increased in the form of interstitials by substitution with Al^3+^. The BVEL analysis suggests an occupation of tetrahedral sites between Na4-Na1 and Na4-Na2 as interstitial positions for the sodium ions (cf. Figure 6). This would be consistent with more efficient 3D ion migration in the structure and overall facilitation of the ion transport.

In the range of *x* = 0.50 − 0.75, where the Na_9_(AlS_4_)(SiS_4_)-structure is stable, the highest conductivities are found. The topology of Na_9_(AlS_4_)(SiS_4_) does not resemble the one of Na_4_SiS_4_ but shows similarities to Na_5_AlS_4_ with a distorted hexagonal packing of [Al/SiS_4_]-[Na2S_6_]-chains. The structure features (migrating) Na ions whose highly distorted coordination polyhedra are connected to other sodium sites mostly via faces and edges, which facilitates ion hopping and approximates 3D diffusion (West and Bruce, 1982). In this sense, the double salt Na_9_(AlS_4_)(SiS_4_) thus shows similarities to the well-known tetragonal LGPS-phase which shows exceptionally high ionic conductivity that is in part attributable to the low energy barrier for lithium diffusion between face-sharing [LiS_4_]-tetrahedra (Hori et al., 2015a; Wang et al., 2015). Additionally, the BVEL analysis of Na_9_(AlS_4_)(SiS_4_) indicates a similar situation to the frustrated energy landscape leading to superionic diffusion in LiTi_2_(PS_4_)_3_ (Di Stefano et al., 2019): The sodium coordination environments are more diverse and the coordination polyhedra more distorted in the double salt Na_9_(AlS_4_)(SiS_4_) compared to the border phases of the substitution series Na_5-*x*_Al_1-*x*_Si_*x*_S_4_. This low local coordination symmetry of sodium and connection of its coordination polyhedra lead to a flat energy landscape for sodium cations in Na_9_(AlS_4_)(SiS_4_), which is beneficial for ion transport.

By further exchanging [AlS_4_]^5−^ anions by [SiS_4_]^4−^ anions in Na_9_(AlS_4_)(SiS_4_), sodium vacancies are introduced. Within the series Na_5-*x*_(AlS_4_)_1-*x*_(SiS_4_)_*x*_ the value *x* = 0.75 (Na_8.5_(AlS_4_)_0.5_(SiS_4_)_1.5_) constitutes the optimum for the observed ionic conductivity of 2.04 · 10^−5^ S cm^−1^ and the lowest activation energy in the series of 0.30 eV as shown in Table S15, reflecting the flattening of the energy landscape proposed by the BVEL calculations. For all other members of the series the activation energies are rather similar within their standard deviation, at around 0.35–0.40 eV (cf. Figure S10). Besides, the pre-factors σ_0_ of the best conducting members of the series exceed the pre-factors of the end members by one to two orders of magnitude (cf. Table S13), although the activation energy is lowered (cf. Figure S10). The pre-factor takes into account the charge carrier density of mobile ions, the entropy of migration, the jump distance and the attempt frequency, among others. Recently, Kraft et al. systematically increased the lattice softness in a series of ionic conductors and noted that a decrease in activation energy is accompanied by a decrease in pre-factor, which is in line with the Meyer-Nedel-rule (Kraft et al., 2017). Accordingly, in cases where this rule applies, possible conductivity improvements via lattice softness engineering are inherently limited. However, Di Stefano et al. (2019) showed for LiTi_2_(PS_4_)_3_ that the highly distorted coordination polyhedra of lithium lead to a frustrated energy landscape, lowering the energy barrier, but increasing the pre-factor due to longer jump distances and a higher entropy for the transition state. In Na_8.5_(AlS_4_)_0.5_(SiS_4_)_1.5_ a similar influence on the pre-factor as in LiTi_2_(PS_4_)_3_ can be inferred due to the flattening of the energy landscape by the highly distorted sodium coordination polyhedra. However, the high pre-factor for the sample *x* = 0.95 compared to the composition with the highest conductivity at *x* = 0.75 could hint to an even more complicated situation in this series of compounds, necessitating further studies on the complex interplay between structural factors and the energetics of ion transport in these systems.

Performance-wise, Na_8.5_(AlS_4_)_0.5_(SiS_4_)_1.5_ with an ionic conductivity of 2.04 · 10^−5^ S cm^−1^ and an activation energy of 0.30 eV is comparable to compounds, such as Na_10_SnP_2_S_12_ (Richards et al., 2016), silicon substituted Na_3_PS_4_ (Tanibata et al., 2014), and HT−NaSi_2_P_3_ (Haffner et al., 2018). As can be concluded from the absence of additional resistances at lower frequencies, this material presumably shows an advantageous microstructure or pressing behavior. Furthermore, the smaller electron affinities of Al^3+^ and Si^4+^ may result in increased electrochemical stability at low potentials compared to thiophosphates, such as Na_3_PS_4_, which will be the subject of future studies.

## 4. Conclusion

We have presented the crystal structures and Na ion conductivities in the novel substitution series Na_5−*x*_Al_1−*x*_Si_*x*_S_4_ with (0 ≤ *x* ≤ 1), containing exclusively low-cost, earth-abundant and lightweight elements. For the best conducting compound Na_8.5_(AlS_4_)_0.5_(SiS_4_)_1.5_ (*x* = 0.75), a relatively high sodium ion conductivity of 2.04 · 10^−5^ S cm^−1^ at room temperature with an activation energy of 0.30 eV was found, putting this material on par with typical sodium solid electrolytes, such as silicon substituted Na_3_PS_4_ (Tanibata et al., 2014) and Na_10_SnP_2_S_12_ (Richards et al., 2016). Our analysis of impedance and BVEL data for the substitution series Na_5-*x*_Al_1-*x*_Si_*x*_S_4_ (0 ≤ *x* ≤ 1) unveils probable sodium ion migration paths and highlights the enhancement of the conductivity by the low local coordination symmetry of the sodium ions flattening out the potential energy landscape and the increase of sodium ion vacancies in Na_8.5_(AlS_4_)_0.5_(SiS_4_)_1.5_. Concluding, the right blend of the cations Al^3+^ and Si^4+^ entails an optimized structure as well as optimal amount of charge carriers for fast sodium ion conduction.

## Data Availability Statement

All datasets generated for this study are included in the article/Supplementary Material. The depositions numbers of cif files in the CCDC database are Na_4_SiS_4_: CCDC 1980423, Na_5_AlS_4_: CCDC 1980422, Na_8.5_AlS_40.5_SiS_41.5_: CCDC 1980426s.

## Author Contributions

SH, A-KH, and BL conceived and designed this study. SH and CHoe conducted the synthesis. SH, A-KH, and CHoe were responsible for measuring SCXRD, PXRD, NMR, and EIS. SH performed the structure determination. CHoc helped in interpretation of crystal structure data. CS performed the calculation and interpretation of BVEL data. A-KH analyzed the measured EIS data. SH, A-KH, and CS wrote the sections of the manuscript. All authors wrote and commented on the manuscript.

### Conflict of Interest

The authors declare that the research was conducted in the absence of any commercial or financial relationships that could be construed as a potential conflict of interest.
