# Supplementary material for: Finding the Right Blend: Interplay Between Structure and Sodium Ion Conductivity in the System Na5AlS4–Na4SiS4
Source: Front Chem. 2020 Feb 18;8:90. doi: 10.3389/fchem.2020.00090 (PMC7040025; doi:10.3389/fchem.2020.00090)
Supplement: Supplementary file 2 [file Data_Sheet_2.PDF]

frontiers

Supplementary Material

1 CRYSTALLOGRAPHIC DATA FOR NA$_5$ALS$_4$

Table S1. Crystallographic data and information for the structure solution and refinement from powder X-ray diffraction data for Na_5AlS_4. Standard deviations are given in parentheses. These data were deposited in the Cambridge Crystallographic Data Centre and were given the deposition number CCDC 1980422.

		Na_5AlS_4
crystal system		orthorhombic
space group		*Pbca*, (Nr. 61)
lattice params.	*a*	12.01930(12) Å
	b	7.05236(7) Å
	c	21.5605(2) Å
V [Å^3]		1827.56(3)
Z		8
calc. density [gcm^{-3}]		1.96398(3)
diffractometer		STOE STADI P, CuK$_{\alpha1}$-radiation
		Debye-Scherrer geometry
temperature [K]		295
absorption coefficient [mm^{-1}]		12.1231(2)
refined 2ϑ region [°]		3 – 90
R_{exp}		6.054
R_p		5.187
R_{wp}		6.604
Goof		1.091
R_{Bragg}		1.949
number of refined params.		60
number of background params.		12

Table S2. Standardized fractional atomic coordinates (Parthé and Gelato (1984)) and isotropic displacement parameters [Å^2] for Na_5AlS_4. Standard deviations are given in parentheses.

Atom	occupation-factor	Wyckoff-position	*x*	*y*	*z*	B_{iso}
S1	1	8*c*	0.3713(4)	0.2046(6)	0.9571(3)	2.58(15)
S2	1	8*c*	0.4477(3)	0.8520(5)	0.3783(3)	2.50(12)
S3	1	8*c*	0.1402(3)	0.2426(6)	0.6222(4)	3.29(12)
S4	1	8*c*	0.6317(4)	0.7610(6)	0.2113(3)	3.4(2)
Al1	1	8*c*	0.0399(3)	0.8312(5)	0.8737(3)	2.70(14)
Na1	1	8*c*	0.3521(5)	0.9080(9)	0.5013(3)	3.8(2)
Na2	1	8*c*	0.2869(4)	0.5531(8)	0.3658(3)	3.6(2)
Na3	1	8*c*	0.5579(6)	0.5658(9)	0.4332(3)	4.6(2)
Na4	1	8*c*	0.5863(5)	0.3809(10)	0.2501(3)	4.3(2)
Na5	1	8*c*	0.8373(5)	0.1078(9)	0.8294(3)	2.9(2)

Table S3: Interatomic distances in Na_5AlS_4. Standard deviations are given in parentheses.
i: $-x+\frac{1}{2}, y-\frac{1}{2}, z$; ii: $-x+1, y-\frac{1}{2}, -z+\frac{3}{2}$; iii: $x, -y+\frac{1}{2}, z+\frac{1}{2}$; iv: $x, -y+\frac{3}{2}, z+\frac{1}{2}$; v: $-x+\frac{1}{2}, -y+1, z+\frac{1}{2}$; vi: $-x+\frac{1}{2}, -y+2, z-\frac{1}{2}$; vii: $-x+1, y+\frac{1}{2}, -z+\frac{1}{2}$; viii: $-x+\frac{3}{2}, -y+1, z-\frac{1}{2}$; ix: $-x+\frac{1}{2}, y+\frac{1}{2}, z$; x: $-x, y-\frac{1}{2}, -z+\frac{3}{2}$; xi: $x-\frac{1}{2}, -y+\frac{1}{2}, -z+1$; xii: $x-\frac{1}{2}, y, -z+\frac{3}{2}$; xiii: $-x+1, y+\frac{1}{2}, -z+\frac{3}{2}$; xiv: $x+\frac{1}{2}, -y+\frac{3}{2}, -z+1$; xv: $x+\frac{1}{2}, y, -z+\frac{1}{2}$; xvi: $x-\frac{1}{2}, -y+\frac{3}{2}, -z+1$; xvii: $-x+\frac{1}{2}, -y+2, z+\frac{1}{2}$; xviii: $-x, y+\frac{1}{2}, -z+\frac{3}{2}$; xix: $x, -y+\frac{3}{2}, z-\frac{1}{2}$; xx: $-x+\frac{1}{2}, -y+1, z-\frac{1}{2}$; xxi: $-x+1, y-\frac{1}{2}, -z+\frac{1}{2}$; xxii: $x, -y+\frac{1}{2}, z-\frac{1}{2}$; xxiii: $x-\frac{1}{2}, y, -z+\frac{1}{2}$; xxiv: $x+\frac{1}{2}, -y+\frac{1}{2}, -z+1$; xxv: $-x+\frac{3}{2}, -y+1, z+\frac{1}{2}$; xxvi: $x+\frac{1}{2}, y, -z+\frac{3}{2}$

Atom1	Atom2	distance [Å]	Atom1	Atom2	distance [Å]
S1	Al1	2.274(8)^{i}		S2	2.241(5)^{xvii}
	Na3	2.696(8)^{ii}		S3	2.255(6)^{xviii}
	Na2	2.864(8)^{iii}		S1	2.274(8)^{ix}
	Na1	2.902(8)^{iv}		Na2	3.084(7)^{iv}
	Na1	2.958(8)^{v}	Na1	S3	2.857(10)^{ix}
	Na3	2.989(8)^{iii}		S1	2.902(8)^{xix}
S2	Al1	2.241(5)^{vi}		S2	2.918(8)
	Na3	2.689(8)		S1	2.958(8)^{xx}
	Na4	2.804(9)^{vii}	Na2	S4	2.822(8)^{xxi}
	Na5	2.805(7)^{viii}		S1	2.864(8)^{xxii}
	Na2	2.873(7)		S2	2.873(7)
	Na1	2.918(8)		S4	2.898(8)^{xxiii}
	Na2	3.168(6)^{ix}		Al1	3.084(7)^{xix}
S3	Al1	2.255(6)^{x}		S2	3.168(6)^{i}
	Na3	2.672(8)^{xi}	Na3	S3	2.672(8)^{xxiv}
	Na5	2.757(7)^{xii}		S2	2.689(8)
	Na5	2.792(8)^{xiii}		S1	2.696(8)^{xiii}
	Na1	2.857(10)^{i}		S1	2.989(8)^{xxii}
	Na4	2.961(10)^{xi}	Na4	S2	2.804(9)^{xxi}
S4	Al1	2.236(9)^{xiv}		S4	2.861(8)
	Na5	2.736(8)^{viii}		S4	2.876(8)^{xxi}
	Na2	2.822(8)^{vii}		S3	2.961(10)^{xxiv}
	Na4	2.861(8)	Na5	S4	2.736(8)^{xxv}
	Na4	2.876(8)^{vii}		S3	2.757(7)^{xxvi}
	Na2	2.898(8)^{xv}		S3	2.792(8)^{ii}
Al1	S4	2.236(9)^{xvi}		S2	2.805(7)^{xxv}

2 CRYSTALLOGRAPHIC DATA FOR NA_4SIS_4

Table S4. Crystallographic data and information for the structure solution and refinement from powder X-ray diffraction data for Na_4SiS_4. Standard deviations are given in parentheses. These data were deposited in the Cambridge Crystallographic Data Centre and were given the deposition number CCDC 1980423.

Na_4SiS_4		
crystal system		orthorhombic
space group		$P2_12_12_1$, (Nr. 19)
lattice params.	a	41.0301(7) Å
	b	8.78409(14) Å
	c	6.88962(12) Å
V [Å^3]		2483.10(7)
Z		12
calc. density [gcm^{-3}]		1.99260(6)
diffractometer		STOE STADI P, MoK$_{\alpha 1}$-radiation<br>Debye-Scherrer geometry
temperature [K]		295
absorption coefficient [mm^{-1}]		1.42581(4)
refined 2ϑ region [°]		2 – 50
R_{exp}		5.390
R_{p}		4.127
R_{wp}		5.377
Goof		0.998
R_{Bragg}		1.825
number of refined params.		109
number of background params.		10

Table S5. Standardized fractional atomic coordinates (Parthé and Gelato (1984)) and isotropic displacement parameters [$Å^2$] for Na_4SiS_4. Standard deviations are given in parentheses.

Atom	occupation-factor	Wyckoff-position	x	y	z	B_{iso}
S1	1	4*a*	0.0315(5)	0.056(3)	0.908(3)	0.8(2)
S2	1	4*a*	0.3654(5)	0.065(3)	0.904(3)	0.8(2)
S3	1	4*a*	0.6993(5)	0.051(3)	0.907(3)	0.8(2)
S4	1	4*a*	0.0621(5)	0.758(3)	0.436(3)	1.54(10)
S5	1	4*a*	0.3963(5)	0.748(3)	0.447(3)	1.54(10)
S6	1	4*a*	0.7289(5)	0.752(3)	0.430(3)	1.54(10)
S7	1	4*a*	0.1346(5)	0.548(3)	0.422(4)	1.5(2)
S8	1	4*a*	0.4662(5)	0.537(3)	0.434(3)	1.5(2)
S9	1	4*a*	0.8019(5)	0.548(3)	0.408(3)	1.5(2)
S10	1	4*a*	0.2185(4)	0.249(3)	0.669(3)	1.37(10)
S11	1	4*a*	0.5436(4)	0.247(3)	0.662(3)	1.37(10)
S12	1	4*a*	0.8813(5)	0.246(3)	0.667(4)	1.37(10)
Si1	1	4*a*	0.2216(6)	0.246(4)	0.962(3)	1.58(10)
Si2	1	4*a*	0.5555(6)	0.248(4)	0.973(4)	1.58(10)
Si3	1	4*a*	0.8868(5)	0.245(4)	0.980(3)	1.58(10)
Na1	1	4*a*	0.2394(6)	0.048(4)	0.386(5)	2.0(3)
Na2	1	4*a*	0.5763(7)	0.045(4)	0.406(5)	2.0(3)
Na3	1	4*a*	0.9051(6)	0.056(4)	0.408(4)	2.0(3)
Na4	1	4*a*	0.2435(6)	0.443(4)	0.381(5)	0.9(3)
Na5	1	4*a*	0.5760(6)	0.452(4)	0.384(5)	0.9(3)
Na6	1	4*a*	0.9166(5)	0.433(3)	0.379(3)	0.9(3)
Na7	1	4*a*	0.1518(7)	0.241(5)	0.256(5)	3.2(2)
Na8	1	4*a*	0.4840(7)	0.242(5)	0.244(5)	3.2(2)
Na9	1	4*a*	0.8199(7)	0.241(5)	0.255(5)	3.2(2)
Na10	1	4*a*	-0.0079(6)	1.036(4)	0.235(4)	3.4(2)
Na11	1	4*a*	0.3330(9)	1.011(5)	0.238(7)	3.4(2)
Na12	1	4*a*	0.6706(8)	0.979(4)	0.270(6)	3.4(2)

Table S6: Interatomic distances in Na_4SiS_4. Standard deviations are given in parentheses.
i: x-$\frac{1}{2}$, -y+$\frac{1}{2}$, -z+2; ii: x-$\frac{1}{2}$, -y+$\frac{1}{2}$, -z+1; iii: x, y-1, z+1; iv: -x+1, y-$\frac{1}{2}$, -z+$\frac{3}{2}$; v: -x+$\frac{1}{2}$, -y, z+$\frac{1}{2}$; vi: x+$\frac{1}{2}$, -y+$\frac{1}{2}$, -z+2; vii: x+$\frac{1}{2}$, -y+$\frac{1}{2}$, -z+1; viii: -x+$\frac{3}{2}$, -y, z+$\frac{1}{2}$; ix: -x+1, y+$\frac{1}{2}$, -z+$\frac{3}{2}$; x: -x+1, y+$\frac{1}{2}$, -z+$\frac{1}{2}$; xi: -x+$\frac{1}{2}$, -y+1, z+$\frac{1}{2}$; xii: x-$\frac{1}{2}$, -y+$\frac{3}{2}$, -z+1; xiii: -x, y-$\frac{1}{2}$, -z+$\frac{1}{2}$; xiv: -x+$\frac{3}{2}$, -y+1, z+$\frac{1}{2}$; xv: x+$\frac{1}{2}$, -y+$\frac{3}{2}$, -z+1; xvi: -x+1, y-$\frac{1}{2}$, -z+$\frac{1}{2}$; xvii: -x+$\frac{1}{2}$, -y, z-$\frac{1}{2}$; xviii: -x+$\frac{3}{2}$, -y, z-$\frac{1}{2}$; xix: -x+$\frac{1}{2}$, -y+1, z-$\frac{1}{2}$; xx: -x+$\frac{3}{2}$, -y+1, z-$\frac{1}{2}$; xxi: x, y+1, z-1; xxii: -x, y+$\frac{1}{2}$, -z+$\frac{1}{2}$;

Atom1	Atom2	distance [Å]	Atom1	Atom2	distance [Å]
S1	Si2	2.15(4)^{i}	Si2	S5	2.05(3)^{iv}
	Na5	2.72(4)^{ii}		S1	2.15(4)^{vi}
	Na10	2.78(4)^{iii}		S8	2.15(4)^{iv}
	Na6	2.80(3)^{iv}		S11	2.20(3)
	Na8	2.83(4)^{ii}		Na6	3.10(4)^{xiv}
	Na8	2.92(5)^{v}		Na3	3.15(5)^{viii}

Continued on next page

Continued from table S6

Atom1	Atom2	distance [Å]	Atom1	Atom2	distance [Å]
S2	Si3	2.05(4)^{i}	Si3	S2	2.05(4)^{vi}
	Na11	2.70(5)^{iii}		S7	2.06(4)^{iv}
	Na9	2.76(4)^{ii}		S12	2.17(3)
	Na6	2.87(3)^{ii}		S4	2.18(3)^{iv}
	Na7	2.96(5)^{v}		Na2	3.01(4)^{viii}
	Na5	2.98(4)^{iv}		Na5	3.14(5)^{xiv}
S3	Si1	2.19(4)^{vi}	Na1	S9	2.64(4)^{xvi}
	Na4	2.69(4)^{vii}		S10	2.77(4)
	Na12	2.84(4)^{iii}		S6	2.95(5)^{ii}
	Na9	2.88(5)^{viii}		S9	3.05(4)^{ii}
	Na7	2.89(4)^{vii}		Si1	3.08(5)^{xvii}
	Na4	2.92(3)^{iv}		S6	3.11(4)^{xvi}
S4	Si3	2.18(3)^{ix}	Na2	S7	2.80(4)^{vii}
	Na6	2.80(3)^{x}		S11	2.84(4)
	Na8	2.84(4)^{xi}		S8	2.92(4)^{xvi}
	Na5	2.89(4)^{xii}		S4	2.93(4)^{vii}
	Na2	2.93(4)^{ii}		Si3	3.01(4)^{xviii}
	Na10	3.18(4)^{xiii}	Na3	S12	2.63(4)
S5	Si2	2.05(3)^{ix}		S7	2.80(4)^{xvi}
	Na3	2.88(4)^{ii}		S8	2.85(3)^{vii}
	Na7	2.90(4)^{xi}		S5	2.88(4)^{vii}
	Na5	3.11(4)^{x}		Si2	3.15(5)^{xviii}
	Na6	3.16(4)^{xii}	Na4	S3	2.69(4)^{ii}
S6	Si1	2.16(3)^{ix}		S10	2.81(4)
	Na1	2.95(5)^{vii}		S3	2.92(3)^{ix}
	Na4	2.95(4)^{x}		S6	2.95(4)^{xvi}
	Na9	3.01(4)^{xiv}		S6	3.04(4)^{xii}
	Na4	3.04(4)^{xv}		Si1	3.13(5)^{xix}
	Na1	3.11(4)^{x}	Na5	S1	2.72(4)^{vii}
S7	Si3	2.06(4)^{ix}		S4	2.89(4)^{xv}
	Na12	2.60(4)^{xii}		S11	2.95(4)
	Na2	2.80(4)^{ii}		S2	2.98(4)^{ix}
	Na3	2.80(4)^{x}		S5	3.11(4)^{xvi}
	Na9	2.80(4)^{x}		Si3	3.14(5)^{xx}
	Na7	3.01(5)	Na6	S4	2.80(3)^{xvi}
S8	Si2	2.15(4)^{ix}		S1	2.80(3)^{ix}
	Na10	2.60(4)^{xv}		S2	2.87(3)^{vii}
	Na3	2.85(3)^{ii}		S12	2.95(3)
	Na2	2.92(4)^{x}		Si2	3.10(4)^{xx}
	Na8	2.99(4)		S5	3.16(4)^{xv}
	Na8	2.99(4)^{x}	Na7	S9	2.79(4)^{xvi}
S9	Si1	2.18(4)^{ix}		S3	2.89(4)^{ii}

Continued on next page

Continued from table S6

Atom1	Atom2	distance [Å]	Atom1	Atom2	distance [Å]
	Na1	2.64(4)^{x}		S5	2.90(4)^{xix}
	Na7	2.79(4)^{x}		S2	2.96(5)^{xvii}
	Na11	2.80(5)^{xv}		S7	3.01(5)
	Na9	2.98(5)	Na8	S1	2.83(4)^{vii}
	Na1	3.05(4)^{vii}		S4	2.84(4)^{xix}
S10	Si1	2.02(3)		S1	2.92(5)^{xvii}
	Na1	2.77(4)		S8	2.99(4)
	Na4	2.81(4)		S8	2.99(4)^{xvi}
	Na12	3.13(4)^{xii}	Na9	S2	2.76(4)^{vii}
	Na11	3.14(5)^{xi}		S7	2.80(4)^{xvi}
S11	Si2	2.20(3)		S3	2.88(5)^{xviii}
	Na2	2.84(4)		S9	2.98(5)
	Na10	2.92(4)^{xi}		S6	3.01(4)^{xx}
	Na10	2.94(4)^{xv}	Na10	S8	2.60(4)^{xii}
	Na5	2.95(4)		S1	2.78(4)^{xxi}
S12	Si3	2.17(3)		S11	2.92(4)^{xix}
	Na3	2.63(4)		S11	2.94(4)^{xii}
	Na6	2.95(3)		S4	3.18(4)^{xxii}
	Na11	2.98(5)^{xv}	Na11	S2	2.70(5)^{xxi}
	Na12	2.99(4)^{xiv}		S9	2.80(5)^{xii}
Si1	S10	2.02(3)		S12	2.98(5)^{xii}
	S6	2.16(3)^{iv}		S10	3.14(5)^{xix}
	S9	2.18(4)^{iv}	Na12	S7	2.60(4)^{xv}
	S3	2.19(4)^{i}		S3	2.84(4)^{xxi}
	Na1	3.08(5)^{v}		S12	2.99(4)^{xx}
	Na4	3.13(5)^{xi}		S10	3.13(4)^{xv}

3 CRYSTALLOGRAPHIC DATA FOR $NA_{8.5}(ALS_4)_{0.5}(SIS_4)_{1.5}$

Table S7. Crystallographic data and information for the structure solution and refinement from powder X-ray diffraction data for $Na_{8.5}(AlS_4)_{0.5}(SiS_4)_{1.5}$. Standard deviations are given in parentheses. These data were deposited in the Cambridge Crystallographic Data Centre and were given the deposition number CCDC 1980426.

		$Na_{16.76(6)}(AlS_4)(SiS_4)_3$
crystal system		monoclinic
space group		*Cc*, (Nr. 9)
lattice params.	*a*	17.5673(6) Å
	b	13.5408(5) Å
	c	14.2543(5) Å
	β	93.3683(13)°
V [Å^3]		3384.9(2)
Z		8
calculated density [gcm^{-3}]		1.982
diffractometer		Bruker D8 Quest (microfocus), MoK_α-radiation, Göbel mirror optics
temperature [K]		295
absorption coefficient [mm^{-1}]		1.382
ϑ-range [°]		2.32 – 27.50
indexing range		$-22 \leq h \leq 22$, $-17 \leq k \leq 17$, $-18 \leq l \leq 18$
number of measured reflexions		66764
number of independent reflexions		7778
number of independent reflexions ($I \geq 2\sigma(I)$)		6660
R_{int}		0.0532
R_σ		0.0302
F(000)		1984
corrections		Lorentz-, polarization-, absorption-effects
absorption correction		multi-scan (SADABS, Krause et al. (2015))
structure solution		direct methods, SHELXS97 (Sheldrick (2008))
structure refinement		least-squares on F^2, SHELXL97 (Sheldrick (2008))
number of free params.		350
Goof		1.040
R values (reflexions satisfying $I \geq 2\sigma(I)$)		$R1 = 0.0348$, $wR2 = 0.0814$
R values (all data)		$R1 = 0.0448$, $wR2 = 0.0863$
residual electron density [$e^-/Å^{-3}$]		0.625/-0.499
twin law		(-1 0 0, 0 -1 0, 0 0 -1)
batch scale factor		0.46

Table S8. Standardized fractional atomic coordinates (Parthé and Gelato (1984)) and equivalent isotropic displacement parameters [Å^2] for $Na_{8.5}(AlS_4)_{0.5}(SiS_4)_{1.5}$. Standard deviations are given in parentheses.

Atom	occupation-factor	Wyckoff-position	*x*	*y*	*z*	$U_{equiv.}$
Na1	1	4*a*	0.0000(2)	0.0160(3)	0.0160(3)	0.0403(8)
Na2	1	4*a*	0.0079(2)	0.2210(4)	0.2467(3)	0.0558(11)
S1	1	4*a*	0.04013(12)	0.0236(2)	0.31978(15)	0.0379(5)
S2	1	4*a*	0.04982(12)	0.2071(2)	0.05134(14)	0.0389(5)
Na3	1	4*a*	0.0627(2)	0.2428(3)	0.4983(4)	0.0610(12)
S3	1	4*a*	0.07158(13)	0.4809(2)	0.24654(15)	0.0352(5)
Na4	1	4*a*	0.1121(3)	0.0854(3)	0.6894(2)	0.0776(14)
S4	1	4*a*	0.14462(12)	0.5680(2)	0.03750(13)	0.0360(5)
Si1	1	4*a*	0.15165(12)	0.07480(14)	0.36658(15)	0.0250(4)
Si2	1	4*a*	0.15633(12)	0.57514(15)	0.19140(14)	0.0255(4)
Na5	0.895(8)	4*a*	0.1588(2)	0.3388(3)	0.1468(2)	0.0488(10)
S5	1	4*a*	0.16361(13)	0.0715(2)	0.51778(14)	0.0450(6)
S6	1	4*a*	0.16354(13)	0.2241(2)	0.3200(2)	0.0492(6)
Na6	0.805(14)	4*a*	0.2040(3)	0.1103(4)	0.1413(3)	0.067(2)
Na7	0.823(7)	4*a*	0.2062(2)	0.4083(2)	0.3655(2)	0.0318(8)
S7	1	4*a*	0.23822(13)	0.0183(2)	0.8137(2)	0.0394(5)
Na8	1	4*a*	0.2465(2)	0.2575(3)	0.5632(3)	0.0551(10)
S8	1	4*a*	0.25849(12)	0.2932(2)	0.00555(15)	0.0384(5)
S9	1	4*a*	0.26822(12)	0.5230(2)	0.23772(15)	0.0409(5)
Na9	1	4*a*	0.3017(2)	0.7230(4)	0.3090(3)	0.0563(11)
Na10	1	4*a*	0.3087(2)	0.4838(3)	0.0561(2)	0.0400(8)
Na11	0.848(7)	4*a*	0.3162(2)	0.1925(3)	0.3152(2)	0.0421(10)
Na12	1	4*a*	0.3179(2)	0.0119(4)	0.4977(3)	0.0756(15)
S10	1	4*a*	0.36523(10)	0.35601(14)	0.44868(12)	0.0274(4)
S11	1	4*a*	0.36644(13)	0.11952(15)	0.13820(14)	0.0355(5)
Si3	1	4*a*	0.37248(11)	0.24120(15)	0.04104(14)	0.0251(4)
S12	1	4*a*	0.38285(11)	0.19002(15)	0.63956(13)	0.0315(4)
Na13	1	4*a*	0.4038(3)	0.03996(10)	0.7780(4)	0.0429(3)
Na14	0.931(5)	4*a*	0.4046(3)	0.43673(10)	0.2774(3)	0.0344(5)
S13	1	4*a*	0.42616(11)	0.8087(2)	0.41727(14)	0.0355(5)
Si4	1	4*a*	0.43545(11)	0.24254(14)	0.51666(13)	0.0200(4)
S14	1	4*a*	0.44125(12)	0.11958(15)	0.41900(13)	0.0336(4)
S15	1	4*a*	0.44322(10)	0.35692(15)	0.10912(12)	0.0280(4)
Na15	0.934(8)	4*a*	0.4911(2)	0.0193(4)	0.0547(4)	0.070(2)
Na16	1	4*a*	0.4949(2)	0.1838(3)	0.2455(2)	0.0511(9)
Na17	0.498(12)	4*a*	0.6027(3)	0.1125(4)	0.4185(4)	0.029(2)
S16	1	4*a*	0.64521(14)	0.2255(2)	0.2408(2)	0.0464(6)
Na18	1	4*a*	0.6571(3)	0.3469(3)	0.4219(3)	0.101(2)

Table S9. Anisotropic displacement coefficients [Å^2] for $Na_{8.5}(AlS_4)_{0.5}(SiS_4)_{1.5}$. U_{ij} is defined by: $U_{ij} = \exp{-2\pi^2[U_{11}(ha^*)^2 + ... + 2U_{21}hka^*b^*]}$. Standard deviations are given in parentheses.

Atom	U_{11}	U_{22}	U_{33}	U_{23}	U_{13}	U_{12}
Na1	0.041(2)	0.038(2)	0.043(2)	0.0120(15)	0.009(2)	0.000(2)
Na2	0.039(2)	0.086(3)	0.041(2)	0.011(2)	-0.003(2)	0.012(2)
S1	0.0258(12)	0.0487(12)	0.0387(11)	-0.0098(9)	-0.0018(9)	-0.0132(9)
S2	0.0284(11)	0.0524(13)	0.0350(10)	-0.0109(10)	-0.0057(9)	0.0035(10)
Na3	0.030(2)	0.053(2)	0.100(3)	0.011(2)	-0.001(2)	0.001(2)
S3	0.0316(12)	0.0352(10)	0.0392(11)	-0.0022(8)	0.0046(8)	-0.0031(9)
Na4	0.119(3)	0.087(3)	0.0269(15)	0.0142(15)	0.008(2)	-0.040(2)
S4	0.0289(10)	0.0538(12)	0.0249(9)	0.0074(8)	-0.0008(7)	0.0046(9)
Si1	0.0206(10)	0.0213(9)	0.0330(10)	-0.0008(8)	0.0009(8)	-0.0006(8)
Si2	0.0203(10)	0.0303(11)	0.0260(9)	0.0073(8)	0.0013(8)	0.0007(8)
Na5	0.047(2)	0.074(2)	0.0256(12)	-0.0047(12)	0.0054(11)	-0.0092(15)
S5	0.0284(11)	0.076(2)	0.0312(10)	-0.0177(10)	0.0022(8)	-0.0026(11)
S6	0.0266(11)	0.0288(10)	0.091(2)	0.0030(11)	-0.0079(11)	0.0033(8)
Na6	0.065(4)	0.086(4)	0.050(3)	-0.018(2)	0.006(2)	0.010(3)
Na7	0.0388(14)	0.0251(13)	0.032(2)	-0.0004(11)	0.0026(11)	0.0020(11)
S7	0.0337(12)	0.0322(10)	0.0539(13)	-0.0034(9)	0.0157(10)	-0.0032(9)
Na8	0.033(2)	0.062(3)	0.070(2)	0.018(2)	0.00(2)	0.012(2)
S8	0.0236(11)	0.0520(13)	0.0390(11)	-0.0186(10)	-0.0022(8)	0.0085(9)
S9	0.0276(12)	0.0585(14)	0.0365(11)	0.0045(10)	0.0017(9)	0.0085(10)
Na9	0.039(2)	0.093(3)	0.036(2)	0.003(2)	-0.0061(15)	-0.026(2)
Na10	0.035(2)	0.040(2)	0.045(2)	0.0006(15)	0.003(2)	0.005(2)
Na11	0.027(2)	0.058(2)	0.040(2)	-0.0115(15)	-0.0123(13)	0.0148(15)
Na12	0.033(2)	0.125(4)	0.069(2)	0.063(2)	-0.002(2)	0.001(2)
S10	0.0272(10)	0.0257(9)	0.0289(9)	-0.0001(7)	-0.0013(7)	0.0009(7)
S11	0.0433(13)	0.0315(10)	0.0313(10)	0.0022(8)	-0.0002(9)	-0.0068(9)
Si3	0.0210(10)	0.0280(10)	0.0261(9)	-0.0030(8)	0.0008(8)	-0.0011(9)
S12	0.0267(10)	0.0409(10)	0.0273(8)	0.0101(8)	0.0049(7)	-0.0030(8)
Na13	0.0519(8)	0.0342(7)	0.0429(7)	0.001(2)	0.0052(6)	0.008(2)
Na14	0.0247(7)	0.0494(9)	0.0287(7)	0.000(2)	-0.0015(5)	-0.002(2)
S13	0.0248(10)	0.0508(12)	0.0307(9)	0.0101(9)	-0.0009(7)	0.0016(9)
Si4	0.0194(10)	0.0236(9)	0.0168(8)	0.0016(7)	0.0001(7)	0.0010(8)
S14	0.0388(12)	0.0319(10)	0.0302(10)	-0.0038(8)	0.0037(8)	0.0041(8)
S15	0.0256(10)	0.0310(9)	0.0272(9)	-0.0072(7)	-0.0016(7)	-0.0052(8)
Na15	0.026(2)	0.074(3)	0.108(4)	-0.053(2)	-0.007(2)	-0.001(2)
Na16	0.042(2)	0.049(2)	0.065(2)	-0.0140(15)	0.0269(15)	-0.0106(13)
Na17	0.031(3)	0.027(3)	0.029(3)	0.002(2)	0.004(2)	0.014(2)
S16	0.0368(13)	0.0221(9)	0.079(2)	-0.0110(10)	-0.0073(11)	0.0018(8)
Na18	0.105(3)	0.056(2)	0.151(4)	0.057(2)	0.085(3)	0.032(2)

Table S10: Interatomic distances in $Na_{8.5}(AlS_4)_{0.5}(SiS_4)_{1.5}$. Standard deviations are given in parentheses. i: x, -y, z-$\frac{1}{2}$; ii: x-$\frac{1}{2}$, y-$\frac{1}{2}$, z; iii: x-$\frac{1}{2}$, -y+$\frac{1}{2}$, z-$\frac{1}{2}$; iv: x, -y, z+$\frac{1}{2}$; v: x, -y+1, z+$\frac{1}{2}$; vi: x-$\frac{1}{2}$, -y+$\frac{1}{2}$, z+$\frac{1}{2}$; vii: x-$\frac{1}{2}$, y+$\frac{1}{2}$, z; viii: x, -y+1, z-$\frac{1}{2}$; ix: x+$\frac{1}{2}$, y+$\frac{1}{2}$, z; x: x+$\frac{1}{2}$, -y+$\frac{1}{2}$, z+$\frac{1}{2}$; xi: x+$\frac{1}{2}$, -y+$\frac{1}{2}$, z-$\frac{1}{2}$; xii: x+$\frac{1}{2}$, y-$\frac{1}{2}$, z;

Atom1	Atom2	distance [Å]	Atom1	Atom2	distance [Å]
Na1	S1	2.756(4)^{i}		Na10	2.808(4)
	S2	2.815(4)		Na18	2.818(5)^{iii}
	S15	2.870(4)^{ii}		Na9	2.954(4)^{viii}
	S10	2.990(4)^{iii}		Na17	3.203(6)^{iii}
	S5	3.105(4)^{i}	S9	Na14	2.696(5)
	Si4	3.474(4)^{iii}		Na10	2.775(4)
	Na4	3.526(5)^{i}		Na9	2.939(5)
	Na10	3.528(2)^{ii}	Na9	S13	2.848(4)
	Na14	3.557(5)^{iii}		S16	2.862(5)^{vii}
	Na18	3.559(5)^{iii}		S8	2.954(4)^{v}
	Na3	3.674(5)^{i}		S12	3.108(4)^{viii}
	Na6	4.205(6)		Si3	3.498(4)^{v}
Na2	S12	2.866(4)^{iii}		Na18	3.513(6)^{vii}
	S6	2.869(4)		Na8	3.590(5)^{viii}
	S1	2.912(5)		Na13	3.716(5)^{viii}
	S2	2.929(4)		Na2	3.779(2)^{ix}
	S13	3.129(5)^{ii}		Na17	4.188(7)^{vii}
	Na5	3.470(6)	Na10	S10	2.866(4)^{viii}
	Si4	3.483(4)^{iii}		S15	2.984(4)
	Na3	3.670(6)		Si3	3.482(4)
	Na13	3.756(6)^{iii}		Na7	3.490(5)^{viii}
	Na9	3.779(2)^{ii}		Na1	3.528(2)^{ix}
	Na6	4.121(7)		Na14	3.546(5)
S1	Si1	2.147(3)		Na8	3.672(5)^{viii}
	Na14	2.692(5)^{ii}		Na17	4.220(6)^{iii}
	Na4	2.739(4)^{i}	Na11	S14	2.758(4)
	Na1	2.756(4)^{iv}		S11	2.895(4)
S2	Si4	2.151(3)^{iii}		S10	3.012(4)
	Na18	2.811(4)^{iii}		S7	3.165(5)^{i}
	Na5	2.896(4)		Na16	3.350(2)
	Na6	3.208(6)		Si4	3.520(4)
	Na17	3.259(6)^{iii}		Na13	3.557(5)^{i}
Na3	S13	2.750(4)^{ii}		Na12	3.570(5)
	S5	2.922(5)	Na12	S11	2.775(4)^{iv}
	S4	2.975(5)^{v}		S14	2.892(5)
	S15	3.020(5)^{vi}		S7	2.928(5)^{i}
	S6	3.192(5)		Na15	3.130(2)^{iv}
	Na8	3.311(2)		S12	3.307(6)

Continued on next page

Continued from table S10

Atom1	Atom2	distance [Å]	Atom1	Atom2	distance [Å]
	Si1	3.389(5)		Na6	3.378(6)^{iv}
	Si3	3.439(4)^{vi}		Na13	3.624(7)^{i}
	Na4	3.527(6)		Na18	3.712(7)^{ii}
	Na15	3.567(7)^{vi}	S10	Si4	2.164(3)
	Na1	3.674(5)^{iv}		Na14	2.799(5)
S3	Si2	2.145(3)		Na10	2.866(4)^{v}
	Na5	2.886(4)		Na1	2.990(4)^{x}
	Na7	2.994(4)	S11	Si3	2.159(3)
	Na13	3.020(6)^{iii}		Na12	2.775(4)^{i}
	Na15	3.049(6)^{vii}		Na16	2.790(4)
	Na17	3.053(6)^{vii}		Na15	2.890(5)
	Na16	3.060(4)^{vii}		Na13	2.985(4)^{i}
Na4	S5	2.665(4)	Si3	S13	2.158(3)^{viii}
	S16	2.717(5)^{vi}		S15	2.191(3)
	S1	2.739(4)^{iv}		Na3	3.439(4)^{xi}
	S7	2.901(5)		Na9	3.498(4)^{viii}
	Na6	3.199(7)^{iv}	S12	Si4	2.149(3)
	S15	3.212(5)^{vi}		Na13	2.841(4)
	Si1	3.370(4)^{iv}		Na2	2.866(4)^{x}
	Na18	3.485(6)^{vi}		Na9	3.108(4)^{v}
	Na1	3.526(5)^{iv}	Na13	S13	2.864(4)^{v}
	Na8	3.840(6)		S11	2.985(4)^{iv}
	Na16	3.851(6)^{vi}		S14	2.998(4)^{iv}
S4	Si2	2.193(3)		S3	3.020(6)^{x}
	Na7	2.756(4)^{viii}		Na16	3.471(4)^{iv}
	Na15	2.802(5)^{vii}		Na11	3.557(5)^{iv}
	Na8	2.974(5)^{viii}		Na12	3.624(7)^{iv}
	Na3	2.975(5)^{viii}		Na15	3.700(8)^{iv}
	Na17	3.040(6)^{iii}		Na9	3.716(5)^{v}
	Na10	3.095(4)		Na2	3.756(6)^{x}
Si1	S6	2.143(3)	Na14	S1	2.692(5)^{ix}
	S7	2.146(3)^{i}		S15	2.752(5)
	S5	2.154(3)		Na1	3.557(5)^{x}
	Na18	3.185(4)^{ii}		Na16	3.813(4)
	Na4	3.370(4)^{i}		Na4	3.934(7)^{xi}
	Na11	3.418(5)	S13	Si3	2.158(3)^{v}
	Na6	3.426(5)		Na3	2.750(4)^{ix}
	Na12	3.480(4)		Na13	2.864(4)^{viii}
Si2	S9	2.155(3)		Na2	3.129(5)^{ix}
	S16	2.167(3)^{vii}		Na15	3.209(6)^{v}
	Na5	3.264(5)	Si4	S2	2.151(3)^{x}
	Na16	3.325(4)^{vii}		S14	2.176(3)

Continued on next page

Continued from table S10

Atom1	Atom2	distance [Å]	Atom1	Atom2	distance [Å]
	Na8	3.365(5)^{viii}		Na1	3.474(4)^{x}
	Na7	3.431(4)		Na2	3.483(4)^{x}
	Na17	3.461(5)^{vii}	S14	Na15	2.801(4)^{iv}
	Na15	3.483(5)^{vii}		Na16	2.835(4)
Na5	S8	2.814(4)		Na17	2.838(6)
	S6	2.913(4)		Na13	2.998(4)^{i}
	Na6	3.196(7)	S15	Na1	2.870(4)^{ix}
	Na7	3.316(4)		Na3	3.020(5)^{xi}
	S9	3.363(5)		Na16	3.145(4)
	Na17	3.408(6)^{iii}		Na4	3.212(5)^{xi}
	Na10	3.586(5)	Na15	S14	2.801(4)^{i}
	Na15	3.991(6)^{vii}		S4	2.802(5)^{xii}
S5	Na12	2.859(5)		S3	3.049(6)^{xii}
	Na8	2.961(5)		Na12	3.130(2)^{i}
	Na6	3.085(6)^{iv}		S13	3.209(6)^{viii}
	Na1	3.105(4)^{iv}		Na17	3.354(7)^{i}
	Na18	3.333(5)^{ii}		Si2	3.483(5)^{xii}
S6	Na7	2.673(4)		Na16	3.513(5)
	Na11	2.721(4)		Na3	3.567(7)^{xi}
	Na6	3.095(6)		Na13	3.700(8)^{i}
Na6	S11	2.859(6)		Na5	3.991(6)^{xii}
	S7	3.044(6)^{i}	Na16	S16	2.705(4)
	S5	3.085(6)^{i}		S3	3.060(4)^{xii}
	Na4	3.199(7)^{i}		Na17	3.171(6)
	Na18	3.238(7)^{iii}		Si2	3.325(4)^{xii}
	Na11	3.270(6)		Na13	3.471(4)^{i}
	S8	3.318(6)		Na4	3.851(6)^{xi}
	Na12	3.378(6)^{i}	Na17	S4	3.040(6)^{x}
Na7	S9	2.674(4)		S3	3.053(6)^{xii}
	S4	2.756(4)^{v}		S16	3.088(6)
	S10	3.053(4)		S8	3.203(6)^{x}
	Na17	3.418(5)^{vii}		S2	3.259(6)^{x}
	Na10	3.490(5)^{v}		Na18	3.315(7)
	Na8	3.518(5)		Na15	3.354(7)^{iv}
	Na11	3.599(5)		Na5	3.408(6)^{x}
	Na14	3.793(6)		Na7	3.418(5)^{xii}
S7	Si1	2.146(3)^{iv}		Si2	3.461(5)^{xii}
	Na18	2.828(4)^{vi}	S16	Si2	2.167(3)^{xii}
	Na12	2.928(5)^{iv}		Na4	2.717(5)^{xi}
	Na13	2.997(6)		Na9	2.862(5)^{xii}
	Na6	3.044(6)^{iv}		Na18	3.058(6)
	Na11	3.165(5)^{iv}		Na8	3.187(5)^{xi}

Continued on next page

Continued from table S10

Atom1	Atom2	distance [Å]	Atom1	Atom2	distance [Å]
Na8	S12	2.730(4)	Na18	S2	2.811(4)^{x}
	S4	2.974(5)^{v}		S8	2.818(5)^{x}
	S10	3.032(4)		S7	2.828(4)^{xi}
	S16	3.187(5)^{vi}		Si1	3.185(4)^{ix}
	Si2	3.365(5)^{v}		Na6	3.238(7)^{x}
	Si4	3.429(4)		S5	3.333(5)^{ix}
	Na9	3.590(5)^{v}		Na4	3.485(6)^{xi}
	Na10	3.672(5)^{v}		Na9	3.513(6)^{xii}
	Na12	3.694(7)		Na1	3.559(5)^{x}
S8	Si3	2.155(3)		Na12	3.712(7)^{ix}

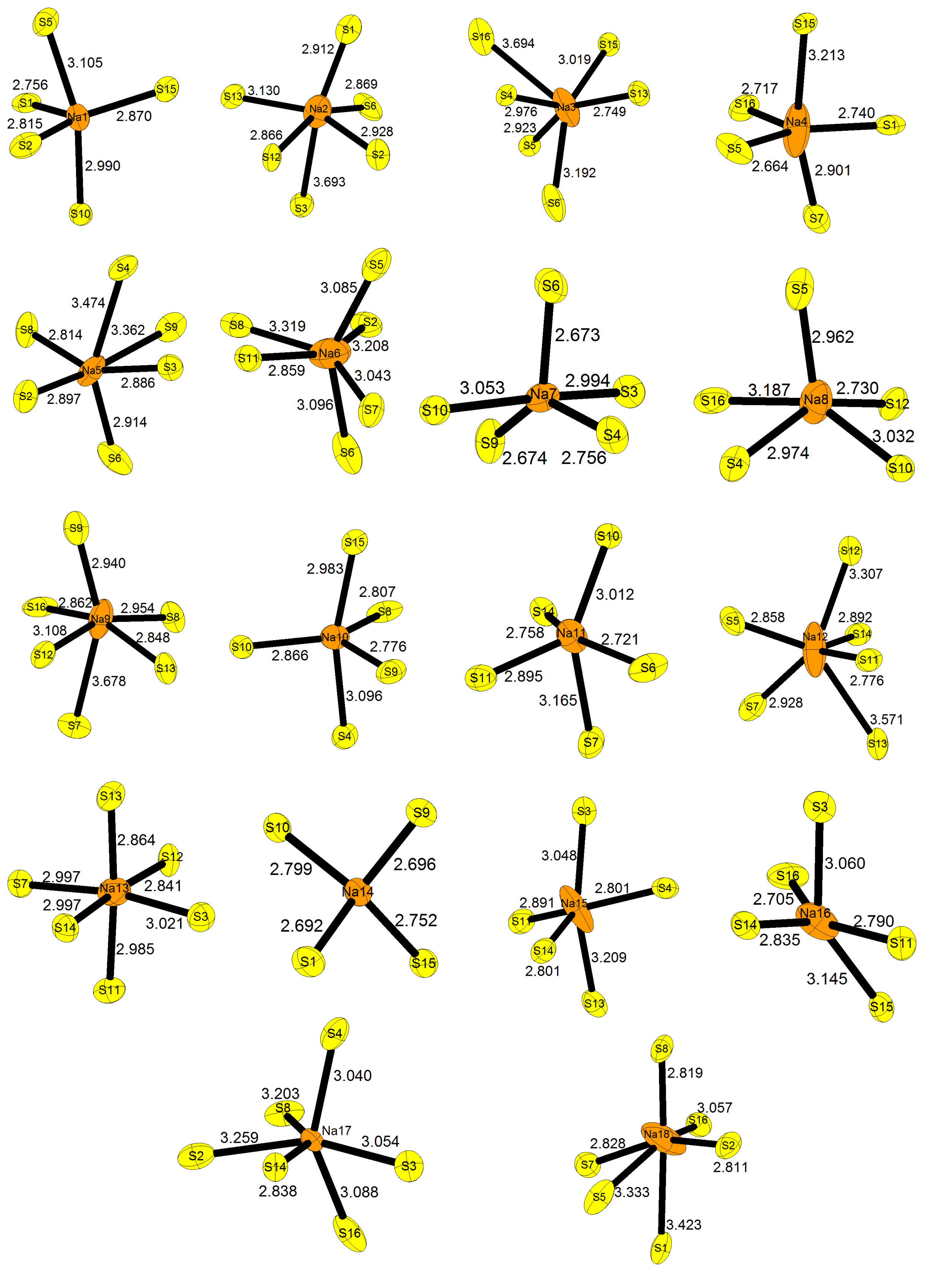

Figure S1. Sodium ion coordination in $Na_{8.5}(AlS_4)_{0.5}(SiS_4)_{1.5}$. Na–S-distances are given in Å. Sodium atoms are depicted in orange, sulfur atoms in yellow. Ellipsoids are drawn at 80% probability.

4 THERMAL ANALYSIS

Due to the occurrence of crystalline side phases in Na_5AlS_4 samples, the synthesis conditions had to be optimized. Therefore, DSC measurements were done for the pseudo-binary border phases Na_4SiS_4 and Na_5AlS_4. Both compounds were synthesized in small (6 mm diameter) sealed carbon coated quartz ampoules at 600 °C suited for DSC measurements and annealed for 3 h. The subsequent DSC measurements are depicted in figure S2. Both compounds show broad endothermic signals during heating at 608 °C (Na_4SiS_4) and 722 °C (Na_5AlS_4), respectively, which are attributed to a complicated multi-step melting process of the materials. In the case of Na_5AlS_4, this is presumed to be a decomposition reaction which also takes place at lower temperatures, albeit at a slower rate, since samples synthesized at 650 °C and above showing increasing amounts of an unknown crystalline side phase. This decomposition behaviour, at least to crystalline side phases, was not observed for Na_4SiS_4. However, the maximum synthesis temperatures were chosen to be below the onset of melting of Na_4SiS_4 at 600 °C, which was suitable for all products.

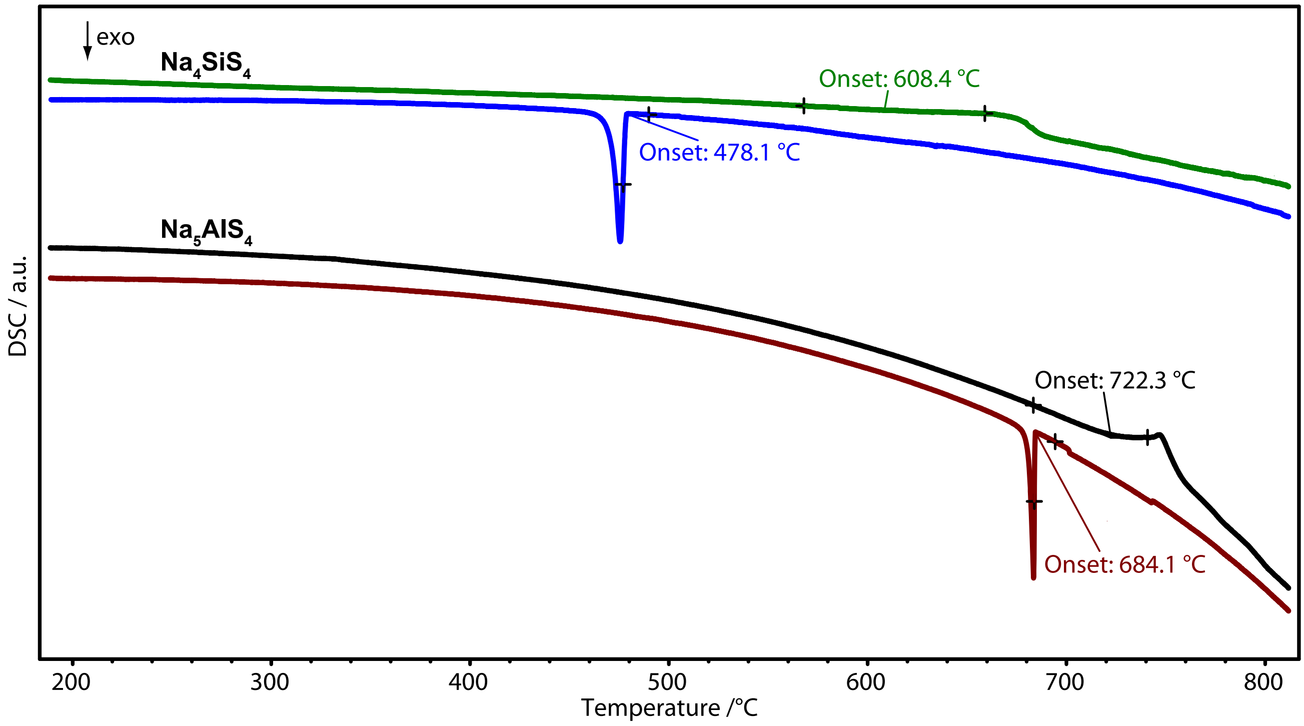

Figure S2. DSC measurement of Na_4SiS_4 and Na_5AlS_4; green and blue lines represent the heating and cooling ramps of Na_4SiS_4, respectively, measured at 5 $Kmin^{-1}$, black and red lines represent the heating and cooling ramps of Na_5AlS_4, respectively, measured at 2 $Kmin^{-1}$.

In addition, we were interested why the only sample showing big single crystals (d $\approx$ 200 μm) was with $x = 75\%$ for $Na_{5-x}Al_{1-x}Si_xS_4$. Therefore, the synthesis conditions were mimicked by heating the precursors to 600 °C for 3 h directly in the DSC machine and measuring the thermal signals during subsequent cooling. The DSC curve (cf. Fig. S3) shows two distinct exothermic signals at 542 °C and 299 °C. The first signal probably corresponds to the crystallization of $Na_{8.5}(AlS_4)_{0.5}(SiS_4)_{1.5}$ from the melt and the latter is in good accordance with the melting point of sodium tetrasulfide (Na_2S_4, m.p.=300 °C) (D'Ans and Lax (1998)). The signal's broad nature also suggests the presence of sodium polysulfides with higher chain lengths. This polysulfide melt could be beneficial for crystal growth by acting as a solvent

for the targeted phase $Na_{8.5}(AlS_4)_{0.5}(SiS_4)_{1.5}$. Since there are no indications of crystalline polysulfides in the PXRD or isolated sodium and sulfur rich areas in SEM/EDX measurements, we suppose that the polysulfide melt forms from unreacted Na_2S and excess S during the reaction and gets gradually consumed with progressing reaction time.

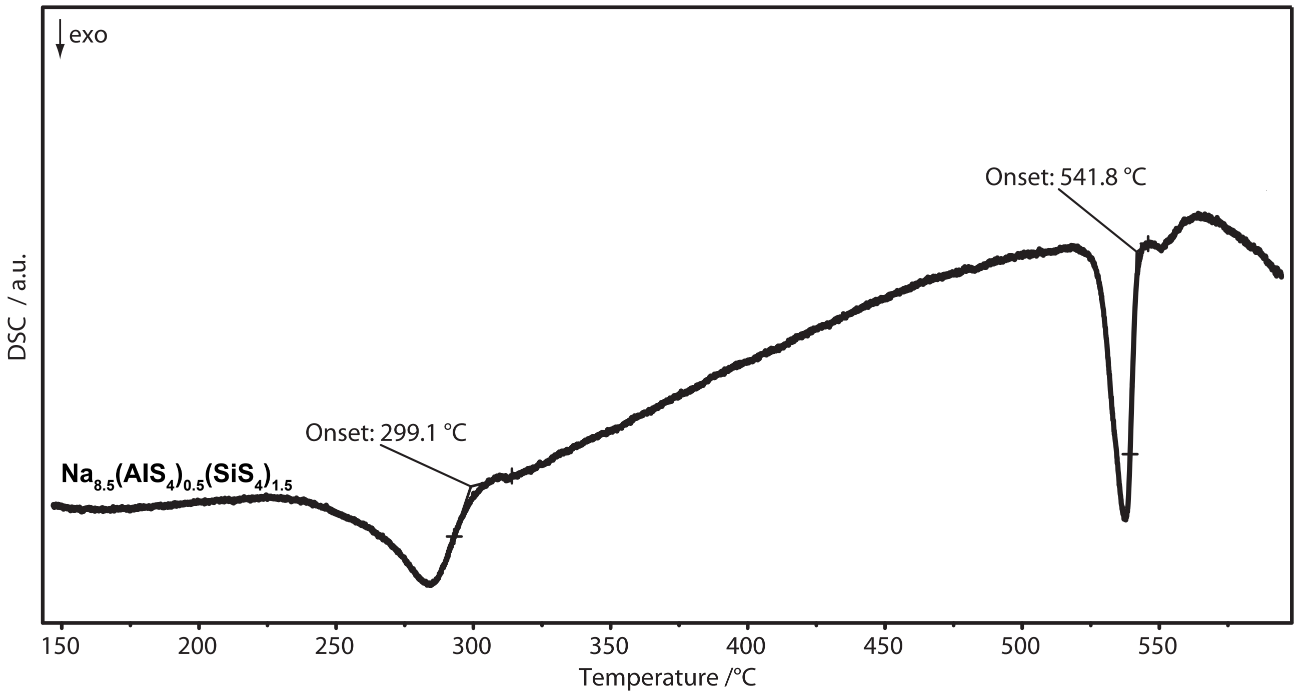

Figure S3. DSC measurement of $Na_{5-x}Al_{1-x}Si_xS_4$, $x = 75\%$; the measurement corresponds to the cooling of the sample at a rate of 5 $Kmin^{-1}$ after annealing it at $600\,°C$ for 3 h.

5 ELECTRON MICROSCOPY

Since the Al/Si occupancy for the single crystal data of $Na_{8.5}Al_{0.5}Si_{1.5}S_8$ ($x = 0.75$) could not be refined because of the similar atomic form factors of the two elements, EDX spectroscopy was used to determine the composition. Table S11 shows the normalized results, averages, the respective standard deviations for O, Na, Al, Si and S, as well as SEM micrographs depicting the positions on the sample. From the SEM micrographs and the values for oxygen it is evident that the sample was partially hydrolyzed on the surface by being in contact with air although exposure time was less than 30 s. While the values for Na and S are systematically too low and show a large spread supposedly due to hydrolysis and the strongly hygroscopic behavior of the sample, Al/Si ratios are within a small error margin and show a slightly lower Al/Si ratio as expected from the weighted precursors, which is in good accordance with the data obtained from SCXRD.

Table S11. Results of EDX measurements for $Na_{8.5}Al_{0.5}Si_{1.5}S_8$ ($x = 0.75$) in atom% normalized to the sum of Al and Si being 4. SEM micrographs depict the positions on the sample.

Position	O	Na	Al	Si	S
SHA089 1	4.693	15.884	0.998	3.002	11.717
SHA089 2	8.492	12.860	1.076	2.924	11.379
SHA089 3	10.782	12.075	0.722	3.278	17.440
SHA089 4	8.844	15.756	0.928	3.072	15.796
SHA089 5	7.596	13.464	1.022	2.978	12.397
SHA089 6	12.085	14.975	0.730	3.270	18.945
SHA089 7	7.760	12.525	1.070	2.930	10.467
SHA089 8	6.326	14.372	0.915	3.085	10.921
SHA089 9	18.805	14.123	0.832	3.168	11.439
SHA089 10	8.225	13.775	0.857	3.143	12.760
SHA089 11	5.089	14.522	0.881	3.119	11.512
SHA089 12	7.019	13.404	0.985	3.015	11.388
SHA089 13	6.663	10.975	1.025	2.975	10.186
SHA089 14	6.982	11.258	0.974	3.026	9.481
SHA089 15	8.892	12.715	1.012	2.988	12.023
SHA089 16	2.589	15.593	0.902	3.098	11.485
average	8	14	0.9	3.1	12
std. deviation	4	2	0.1	0.1	3

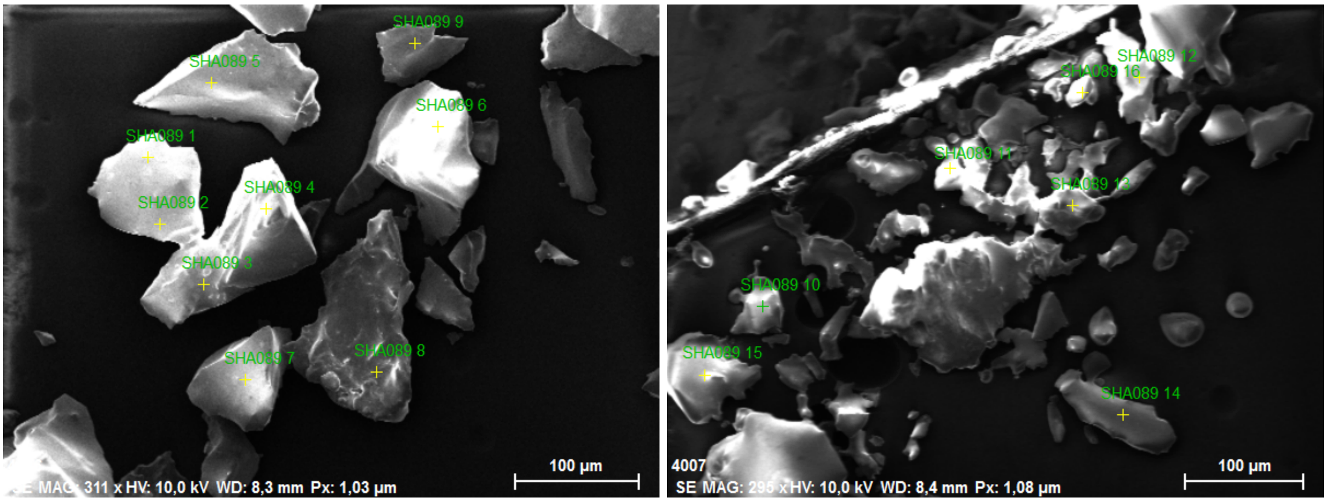

5.1 BVEL calculations

5.1.1 Description of BVEL calculations

In this section we describe the general procedure of calculating bond valence sums and bond valence energy landscapes using the program 3DBVSMAPPER. We also discuss the limitations of this method.
In practice, the program 3DBVSMAPPER executes the following procedure:
(i) Removal of all atoms of low occupancy ($\mathrm{SOF} < 0.05$).
(ii) Removal of the tested ion (here Na^+).
(iii) Creation of equal size voxel points across the unit cell (here: 0.2 Å).
(iv) Calculation of the BVEL at each point in real space.
(v) Analysis of the volumetric data: calculation of the global minimum energy $\mathrm{E}_{\mathrm{min}}^{\mathrm{global}}$, the minimum energy within the infinitely connected pathway $\mathrm{E}_{\mathrm{min}}^{\mathrm{path}}$ and the energy $\mathrm{E}_{\mathrm{mig}}^{\mathrm{path}}$, at which a infinitely connected pathway is formed.
(vi) Generation of a periodic grid file (*.grd).

The output file of *.grd extension can be used as a volumetric data input for VESTA (Momma and Izumi (2011)). Although the BV approach used in this work provides quite accurate insights into ion migration pathways in possible crystalline solid ion conductors, some factors are not considered in this method. In DFT and molecular dynamics calculations the relaxation of the anionic lattice during ion migration through a bottleneck is usually considered. This relaxation, however, is lowering the potential energy of the transition sate and therefore lowering $\mathrm{E}_{\mathrm{mig}}$, compared to the energy of the transition state calculated by the BV approach. Additionally, coulombic repulsion of Na^+–Na^+ is not included in this model, since all tested ions are removed from the structure before calculation. This can lead to underestimating the energy at real space points of higher test ion probability (e.g. along the conduction pathway), since the BV calculated energy landscape mimics a migration network for one test ion per unit cell. This is less important for materials with low concentrations of the migrating ion but plays a more crucial role in materials with high mobile ion concentrations. Pathways might be clogged by repulsion of ions with the same charge, may they be of the same or different kind, thus increasing $\mathrm{E}_{\mathrm{mig}}$. Additionally, the BV method is restricted to mostly ionic compounds, excluding compounds with a more covalent bonding character and metals. Despite these methodological drawbacks, calculated bond valence energy landscapes provide fast and computationally cheap access for investigating ion migration pathways in crystalline (ionic) solids.

5.2 BVEL calculations of Na_5AlS_4

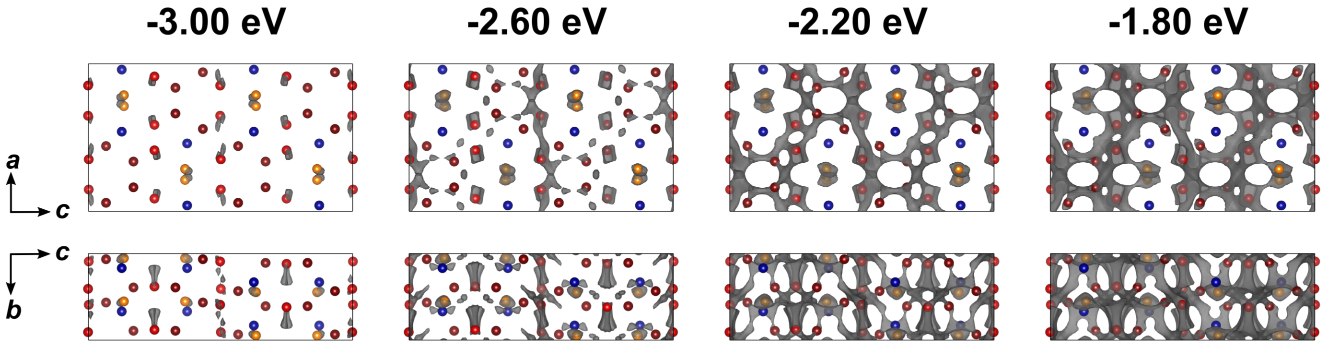

Figure S4. Crystal structure of Na_5AlS_4 with Na atoms drawn in red (migrating ions) and orange (isolated ions), Al atoms drawn in blue and sulfur atoms depicted in yellow. Bond valence energy landscape at different isoenergy values are drawn in grey.

5.3 BVEL calculations of Na_4SiS_4

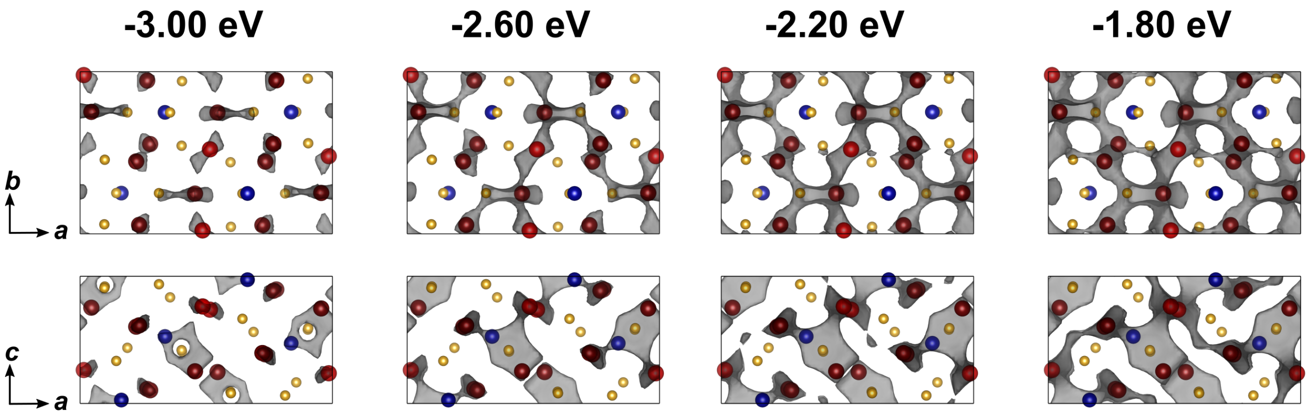

Figure S5. Crystal structure of Na_4SiS_4 with Na atoms drawn in red, Si atoms drawn in blue and sulfur depicted in yellow. Bond valence energy landscape at different isoenergy values are drawn in grey.

5.4 BVEL calculations of $Na_9(AlS_4)(SiS_4)$

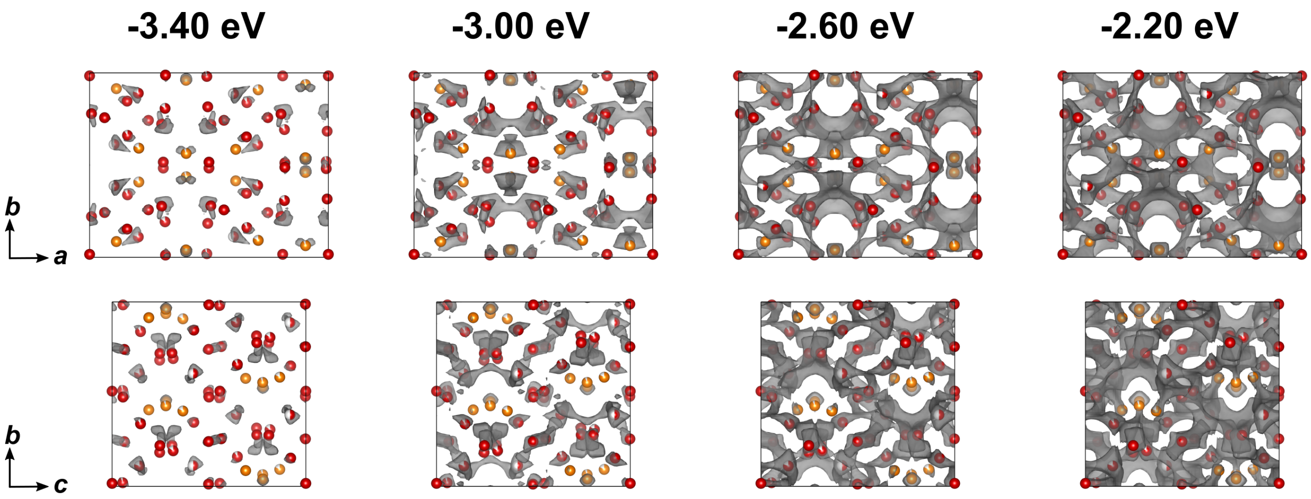

Figure S6. Crystal structure of $Na_9(AlS_4)(SiS_4)$ with Na atoms drawn in red (migrating ions) and orange (isolated ions). Bond valence energy landscape at different isoenergy values are drawn in grey.

6 ELECTROCHEMICAL CHARACTERIZATION

6.1 Galvanostatic polarization measurements

A transference number t_{ion} of 0.9998 of an representative sample ($Na_{5-x}Al_{1-x}Si_xS_4$ with $x = 0.05$) was determined by direct current galvanostatic polarization measurements using blocking-electrodes (stain less steel). The material is thus a mainly ionic conductor (Maier (2004); Harm et al. (2019)).

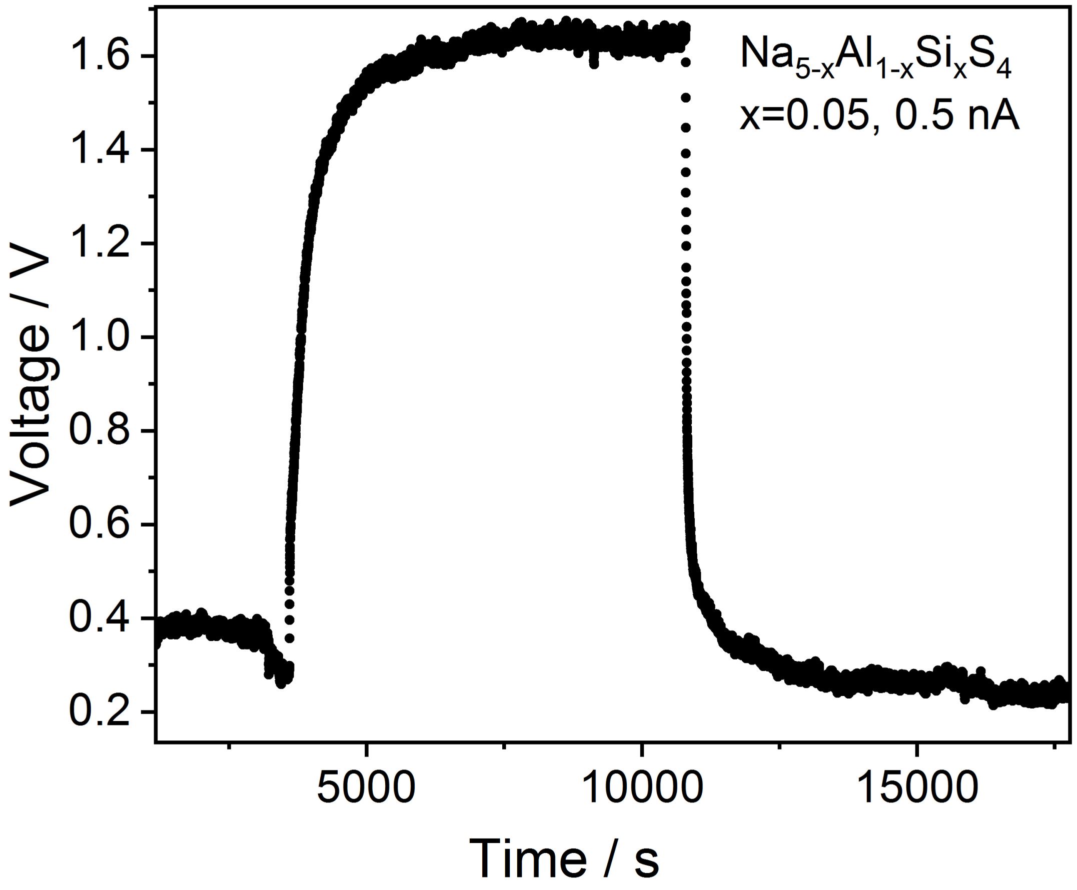

Figure S7. Galvanostatic polarization measurement of $Na_{5-x}Al_{1-x}Si_xS_4$ with $x = 0.05$ with a current of 0.5 nA shows an electronic conductivity of $6x10^{-11} S\,cm^{-1}$ and a transference number of 0.9998. The material is clearly a mainly ionic conducting material.

7 ELECTROCHEMICAL IMPEDANCE SPECTROSCOPY

Table S12. Ionic conductivities calculated from R_1 and R_{Tot} (=R_1+R_2) and the respective capacitances of representative measurements shown in Figure S8 at 25 °C of $Na_{5-x}Al_{1-x}Si_xS_4$ with ($0 \leq x \leq 1$). The electrode area was 0.20 cm^2 and the thickness of the pellets in the range of 0.35-0.75 mm.

x	σ_{R1} / $S\,cm^{-1}$	σ_{RTot} / $S\,cm^{-1}$	$Ceff_1$ / F	CPE_1	α_1	$Ceff_2$ / F	CPE_2	α_2
0.00	4.27E-7	1.52E-7	2.26E-10	3.11E-10	0.96	1.75E-8	8.46E-8	0.59
0.025	3.72E-7	2.18E-7	1.86E-10	3.15E-10	0.94	2.35E-8	1.54E-7	0.59
0.05	6.41E-7	2.86E-7	1.96E-10	2.77E-10	0.96	2.02E-8	1.63E-7	0.50
0.10	4.81E-7	1.27E-7	2.19E-10	5.55E-10	0.90	3.44E-7	6.29E-7	0.42
0.25	1.82E-6	5.47E-7	2.22E-10	-	-	2.48E-8	8.48E-8	0.76
0.50	5.19E-6	2.76E-6	2.06E-10	-	-	5.11E-8	4.53E-7	0.63
0.625	7.14E-6	-	2.41E-10	3.63E-10	0.97	-	-	-
0.75	2.78E-5	-	1.86E-10	-	-	-	-	-
0.90	2.31E-6	1.46E-6	2.00E-10	-	-	4.56E-8	1.53E-7	0.78
0.95	5.83E-6	7.64E-7	2.75E-10	-	-	2.81E-8	1.59E-7	0.67
1.00	2.85E-7	-	1.58E-10	-	-	-	-	-

Table S13. Pellet density of $Na_{5-x}Al_{1-x}Si_xS_4$ with ($0 \leq x \leq 1$) samples shown in Figure S8 and their activation energies with prefactor σ_0 averaged over several temperature cycles.

x	pellet density	$E_a(R1)$ / eV	standard deviation (E_a)	σ_0 / $KS\,cm^{-1}$	standard deviation (σ_0)
0.00	0.87	0.35	0.01	118	39
0.025	0.81	0.36	0.02	154	99
0.05	0.91	0.37	0.01	288	74
0.10	0.86	0.35	0.01	116	26
0.25	0.80	0.37	0.003	1059	154
0.50	0.87	0.35	0.01	1106	278
0.625	0.87	0.36	0.01	2954	681
0.75	0.88	0.31	0.001	1684	59
0.90	0.88	0.34	0.01	394	86
0.95	0.80	0.40	0.01	4389	1603
1.00	0.76	0.36	0.01	78	24

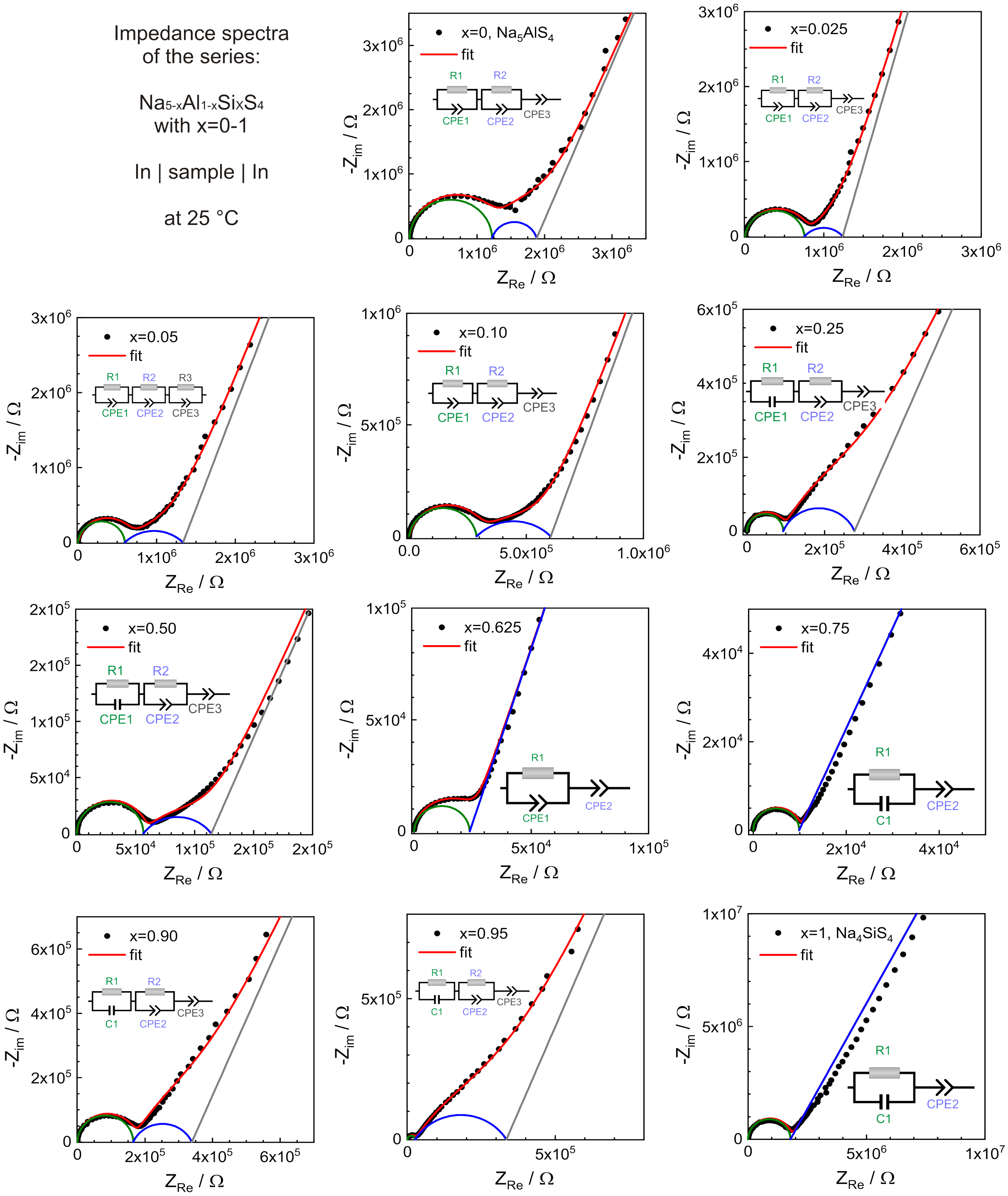

Figure S8. For each member of the series $Na_{5-x}Al_{1-x}Si_xS_4$ with $(0 \leq x \leq 1)$ a representative impedance spectrum with fit and equivalent circuit model at 25 °C is given. The according values for conductivity, capacitance, activation energy and the prefactor are given in TableS12 and TableS13. For x = 0.625,0.75 and 1.00 the inclusion of a low frequency semicircle into the model leads to overfitting. Thus, the best fitting model consists only of one RC- or RCPE-element in series to an CPE.

Table S14. Ionic conductivity data calculated from R_{Tot} and averaged over all samples measured for each member of the series $Na_{5-x}Al_{1-x}Si_xS_4$ with $(0 \leq x \leq 1)$ at $25\,°C$. The activation energies $\sigma(R_{Tot})$ were averaged over all samples and several temperature cycles for each sample. The electrode area was 0.20 cm^2 and the thickness of the pellet in the range of 0.35-0.75 mm.

x	$\sigma_{R_{Tot}}$ / Scm^{-1}	Standard deviation and error	$E_a(R_2)$ / eV	Standard deviation
0.00	1.68E-07	1.91E-08	0.38	0.09
0.025	2.01E-07	1.35E-08	0.50	0.04
0.05	2.63E-07	3.35E-08	0.42	0.04
0.10	1.31E-07	3.22E-08	0.46	0.04
0.25	7.49E-07	5.04E-07	0.39	0.04
0.50	1.81E-06	3.63E-07	0.37	0.08
0.625	-	-	-	-
0.75	-	-	-	-
0.90	8.93E-07	3.34E-07	0.36	0.04
0.95	5.46E-07	8.57E-08	0.63	0.03
1.00	-	-	-	-

Table S15. Ionic conductivity data calculated from R_1 and averaged over all samples measured for each member of the series $Na_{5-x}Al_{1-x}Si_xS_4$ with $(0 \leq x \leq 1)$ at $25\,°C$. The activation energies $\sigma(R_1)$ were averaged over all samples and several temperature cycles for each sample. The electrode area was 0.20 cm^2 and the thickness of the pellet in the range of 0.35-0.75 mm.

x	σ_{R1} / Scm^{-1}	Standard deviation and error	E_a / eV	Standard deviation
0.00	3.20E-07	3.12E-08	0.35	0.01
0.025	3.07E-07	3.65E-08	0.39	0.02
0.05	6.42E-07	8.99E-08	0.37	0.02
0.10	1.29E-06	8.20E-08	0.37	0.01
0.25	2.06E-06	1.38E-07	0.38	0.01
0.50	7.04E-06	4.23E-07	0.36	0.01
0.625	6.44E-06	2.69E-07	0.33	0.02
0.75	2.04E-05	1.32E-06	0.31	0.01
0.90	1.89E-06	2.04E-07	0.33	0.01
0.95	4.49E-06	1.16E-06	0.37	0.01
1.00	1.64E-07	4.64E-08	0.38	0.01

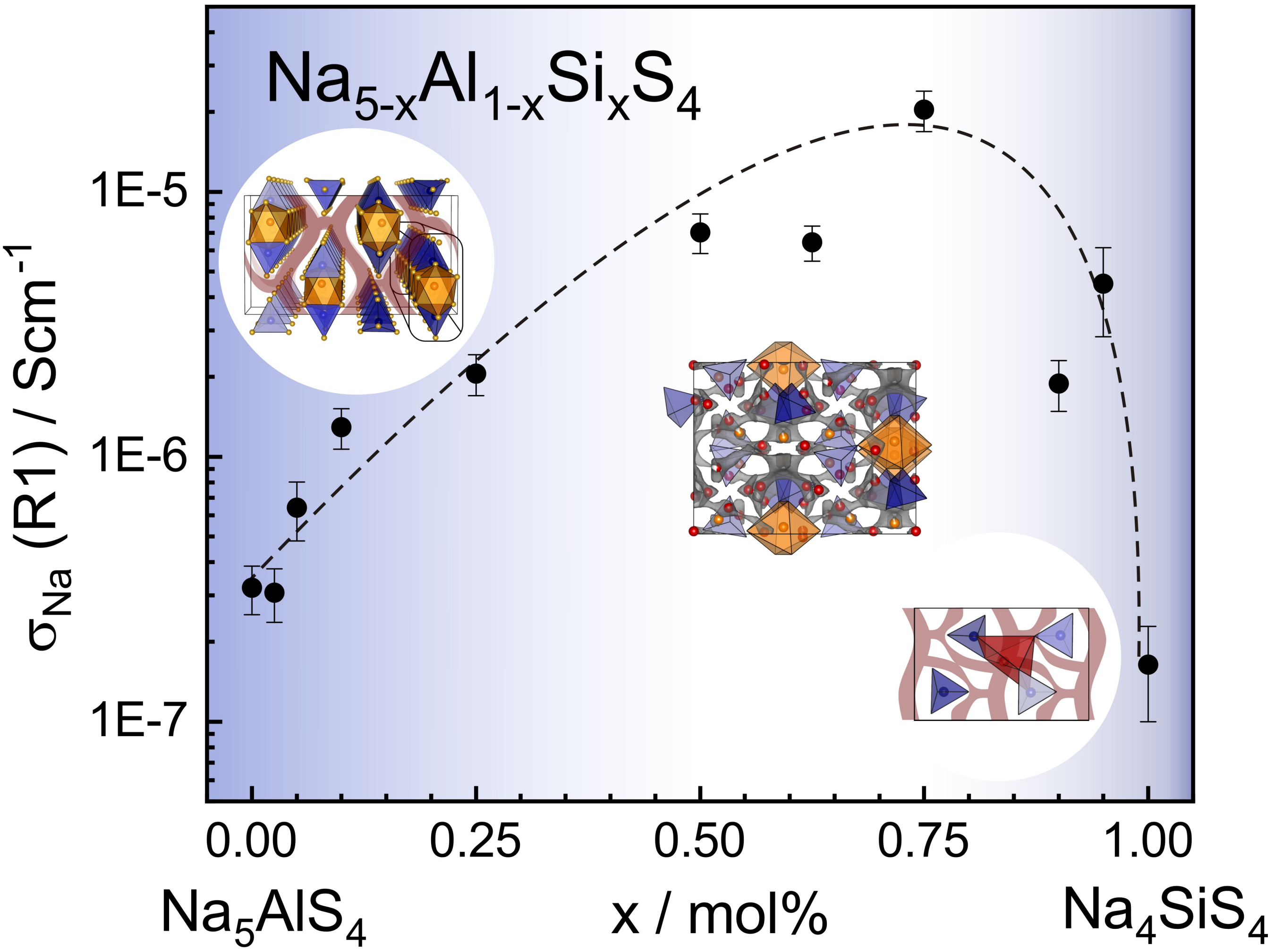

Figure S9. Ionic conductivities calculated exclusively from the resistance of the high frequency semicircle (R1) excluding resistive processes occurring for some member of in $Na_{5-x}Al_{1-x}Si_xS_4$ with $(0 \leq x \leq 1)$. The error bars denote the error and the standard deviation of the values.

7.1 Activation Energies

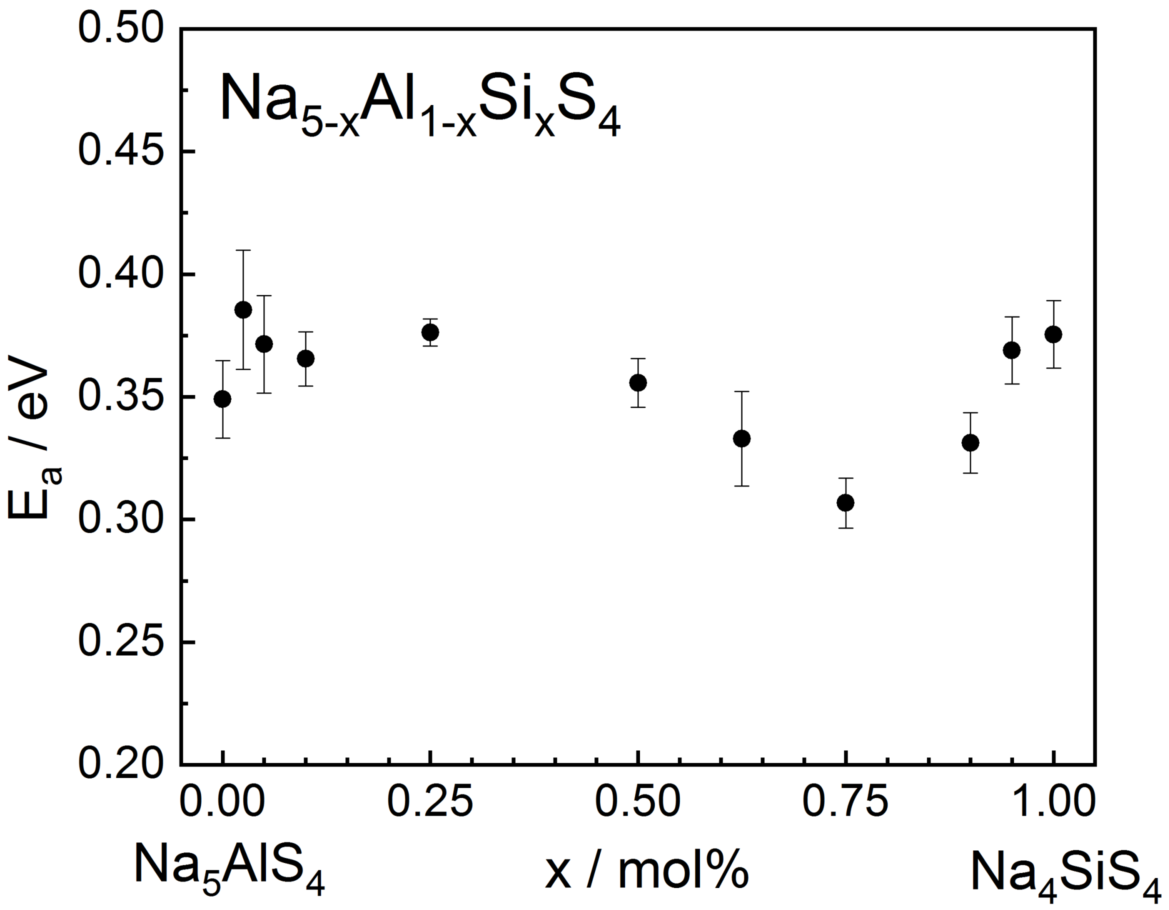

Figure S10. The measured activation energies range from 0.30-0.38 eV for the whole substitution range of $Na_{5-x}Al_{1-x}Si_xS_4$ with $(0 \leq x \leq 1)$. The lowest activation energy can be found in the double salt at x=0.75. The error bars denote the error and the standard deviation of the values.

REFERENCES

D'Ans and Lax (1998). *D'Ans-Lax Taschenbuch für Chemiker und Physiker* (Berlin, Heidelberg: Springer), 3 edn. doi:10.1007/978-3-642-58842-6

Harm, S., Hatz, A.-K., Moudrakovski, I., Eger, R., Kuhn, A., Hoch, C., et al. (2019). Lesson Learned from NMR: Characterization and Ionic Conductivity of LGPS-like Li_7SiPS_8. *Chem. Mater.* 31, 1280–1288. doi:10.1021/acs.chemmater.8b04051

Krause, L., Herbst-Irmer, R., Sheldrick, G. M., and Stalke, D. (2015). Comparison of silver and molybdenum microfocus X-ray sources for single-crystal structure determination. *J. Appl. Crystallogr.* 48, 3–10. doi:10.1107/S1600576714022985

Maier, J. (2004). *Physical Chemistry of Ionic Materials* (Chichester: John Wiley & Sons)

Momma, K. and Izumi, F. (2011). *VESTA3* for three-dimensional visualization of crystal, volumetric and morphology data. *J. Appl. Cryst.* 44, 1272–1276. doi:10.1107/S0021889811038970

Parthé, E. and Gelato, L. M. (1984). The standardization of inorganic crystal-structure data. *Acta Crystallogr.* A40, 169–183. doi:10.1107/S0108767384000416

Sheldrick, G. M. (2008). A short history of SHELX. *Acta Crystallogr.* A64, 112–122. doi:10.1107/S0108767307043930
